# Relation between mitochondrial DNA hyperdiversity, mutation rate and mitochondrial genome evolution in *Melarhaphe neritoides* (Gastropoda: Littorinidae) and other Caenogastropoda

**DOI:** 10.1038/s41598-018-36428-7

**Published:** 2018-12-19

**Authors:** Séverine Fourdrilis, Antonio M. de Frias Martins, Thierry Backeljau

**Affiliations:** 1 Royal Belgian Institute of Natural Sciences, Rue Vautier 29, B-1000 Brussels, Belgium; 2 CIBIO, Centro de Investigação em Biodiversidade e Recursos Genéticos, InBIO Laboratório Associado, Pólo dos Açores, Departamento de Biologia da Universidade dos Açores, Campus de Ponta Delgada, Apartado 1422, 9501-801 Ponta Delgada, Açores Portugal; 3 Evolutionary Ecology Group, University of Antwerp, Universiteitplein 1, B-2610 Antwerp, Belgium

## Abstract

Mitochondrial DNA hyperdiversity is primarily caused by high mutation rates (*µ*) and has potential implications for mitogenome architecture and evolution. In the hyperdiverse mtDNA of *Melarhaphe neritoides* (Gastropoda: Littorinidae), high mutational pressure generates unusually large amounts of synonymous variation, which is expected to (1) promote changes in synonymous codon usage, (2) reflect selection at synonymous sites, (3) increase mtDNA recombination and gene rearrangement, and (4) be correlated with high mtDNA substitution rates. The mitogenome of *M. neritoides* was sequenced, compared to closely related littorinids and put in the phylogenetic context of Caenogastropoda, to assess the influence of mtDNA hyperdiversity and high *µ* on gene content and gene order. Most mitogenome features are in line with the trend in Mollusca, except for the atypical secondary structure of the methionine transfer RNA lacking the TΨC-loop. Therefore, mtDNA hyperdiversity and high *µ* in *M. neritoides* do not seem to affect its mitogenome architecture. Synonymous sites are under positive selection, which adds to the growing evidence of non-neutral evolution at synonymous sites. Under such non-neutrality, substitution rate involves neutral and non-neutral substitutions, and high *µ* is not necessarily associated with high substitution rate, thus explaining that, unlike high *µ*, a high substitution rate is associated with gene order rearrangement.

## Introduction

*Melarhaphe neritoides* (Linnaeus, 1758) is a littorinid periwinkle that shows mitochondrial DNA (mtDNA) hyperdiversity, i.e. its selectively neutral nucleotide diversity is above the threshold of 5% (see^1^ for this definition and threshold), for the cytochrome oxidase c subunit I (*cox1*) and cytochrome b (*cob*) genes (*π*_*syn*_ = 7.4 and 6.4% respectively). This is probably due to an extremely high mtDNA mutation rate (*µ* = 5.82 × 10^−5^ per site per year at the *cox1* locus)^2^. Across eukaryotic phyla, such mtDNA hyperdiversity may be more common than currently appreciated^2^.

Because of its alleged association with high mutation and substitution rates, mtDNA hyperdiversity is expected to affect mitogenome architecture (i.e. gene content and gene order) and evolution in four ways. Firstly, synonymous variation is generated by mutational pressure and is a substrate for genetic-code alteration^3^. As such, we hypothesise that the unusually large amount of synonymous variation in hyperdiverse mtDNA promotes genetic-code alterations and may be associated with changes in synonymous codon usage.

Secondly, because DNA hyperdiversity, mitochondrial or nuclear, is estimated from synonymous substitutions that are assumed to be neutral, it represents neutral polymorphism, which results from the balance between mutation pressure, genetic drift and gene flow. Yet, synonymous substitutions might be under selection pressures, because a change of a nucleotide produces different, though synonymous, codons, some of which are more accurately and/or efficiently translated than others^4^. For example, weak selection for codon usage bias leading to non-neutral synonymous sites has been observed in the nematode *Caenorhabditis remanei*^5^, while strong purifying selection on synonymous sites has been reported in *Drosophila melanogaster*^6^. So, contrary to the mainstream idea, selection can act upon synonymous variation^1^. Thus, the unusually large amount of synonymous variation in hyperdiverse mtDNA, may contribute to adaptation and may result in significant selection pressures for mitonuclear coevolution favouring compatibility between nuclear- and mtDNA-encoded proteins, and hence may play a major role in mitogenome evolution^7^. Conversely, the crucial metabolic functions of mitochondrial protein-coding genes (PCGs) in energy production and in mitonuclear coevolution constrain adaptive mtDNA variation^7^, and hence may limit the impact of mtDNA hyperdiversity on mitogenome architecture and evolution. So, in order to better understand the potential impact of mtDNA hyperdiversity on mtDNA evolution, it is important to explore the balance between the power of mtDNA hyperdiversity to produce adaptive variation and the limits imposed on such variation by the metabolic constraints of mtDNA.

Thirdly, an increase in mtDNA mutation rate may lead to an increase in the rate of mtDNA recombination^8^. Recombination in animal mtDNA promotes variation in mitogenome architecture by inducing gene deletions and rearrangements^9,10^, and increases genetic diversity by spreading new recombinant mitogenomes^11^; thence, we hypothesise that the very high mutation rate in hyperdiverse mtDNA may promote recombination and gene order rearrangement. Conversely, high polymorphism in DNA might inhibit homologous recombination^12^, due to a reduced efficiency of enzymes catalysing annealing and recombination between related, but more strongly diverged DNA sequences^10^. Recombination in animal mitogenomes is rare but occurs in e.g. arthropods, bivalve and cephalopod molluscs, cephalochordates, nematodes, and vertebrates including human^13,14^. Recombination of mtDNA was also suggested, but not tested, in the gastropod *Lottia digitalis*^15^. Gene rearrangements are infrequent in metazoan mitogenomes^16^, except in molluscs, where they are unusually frequent^15–18^, and may even occur within family-level taxa such as among genera of the gastropod family Vermetidae^19^.

Finally, mutations that are selected and fixed, accumulate over time in an evolving lineage, and contribute to substitution rates. High mtDNA substitution rates and high rates of mitochondrial genome rearrangements are positively associated in invertebrates^8,20–22^. However, this positive association is not straightforward^18,23^ and needs further corroboration. For example, there is no clear indication of a particularly high mtDNA substitution rate in *M. neritoides* and whether it is associated to a gene order that differs from that of other littorinids. The mtDNA substitution rate in *M. neritoides* is not known, and no phylomitogenomic data are available for *M. neritoides*. The substitution rate in *M. neritoides*, as estimated from the ultrametric species-level phylogeny of the subfamily Littorininae (mtDNA and nDNA combined) presented by Reid *et al*.^24^, is according to our calculations 6.11 × 10^−3^ substitutions per site per million years, which is of the same order of magnitude as the substitution rates in the other Littorininae included by Reid *et al*.^24^. However, unpublished phylogenies based on the single mtDNA *cox1* and *rrnS* genes extracted from the aforementioned study David Reid, in litt.^24^ shows *M. neritoides* as terminal branch of an intermediate length within the Littorininae, yet longer than in the nuclear *28S* phylogeny, indicating that the mitochondrial substitution rate is likely higher when mtDNA data are not combined with nuclear data.

The expected implications of mtDNA hyperdiversity for mitogenome architecture and evolution lead to several questions: (1) Does the extremely high mutational pressure on synonymous variation underlying mtDNA hyperdiversity affect mitogenome architecture? (2) Does non-neutral synonymous variation affect mitogenome architecture? (3) Do elevated substitution rates affect mitogenome architecture? We explore these questions in *M. neritoides*, the only mollusc in which hitherto mtDNA hyperdiversity has been associated with an extremely high mutational pressure. Hence, we here assess whether this mtDNA hyperdiversity is associated with changes in base composition, codon usage, tRNA structure, gene order and selection on protein-coding genes. To this end, we conduct a comparative mitogenomic analysis of *M. neritoides* and the three other littorinid species whose mitogenomes have been sequenced, viz. *Littorina fabalis*, *Littorina obtusata* and *Littorina saxatilis* (hereafter referred to as “*Littorina* sp.”)^25^, and extend this analysis to other Caenogastropoda. *Melarhaphe neritoides* is a caenogastropod that belongs to non-latrogastropod Hypsogastropoda, within which the family Naticidae is the closest phylogenetic relative, Hydrobiidae, Pomatiopsidae and Provannidae are more distant, and Cassidae, Cypraeidae, Ranellidae and Strombidae are the most distant relatives^26,27^.

## Results and Discussion

### Mitogenome organisation and composition

**Figure 1:**
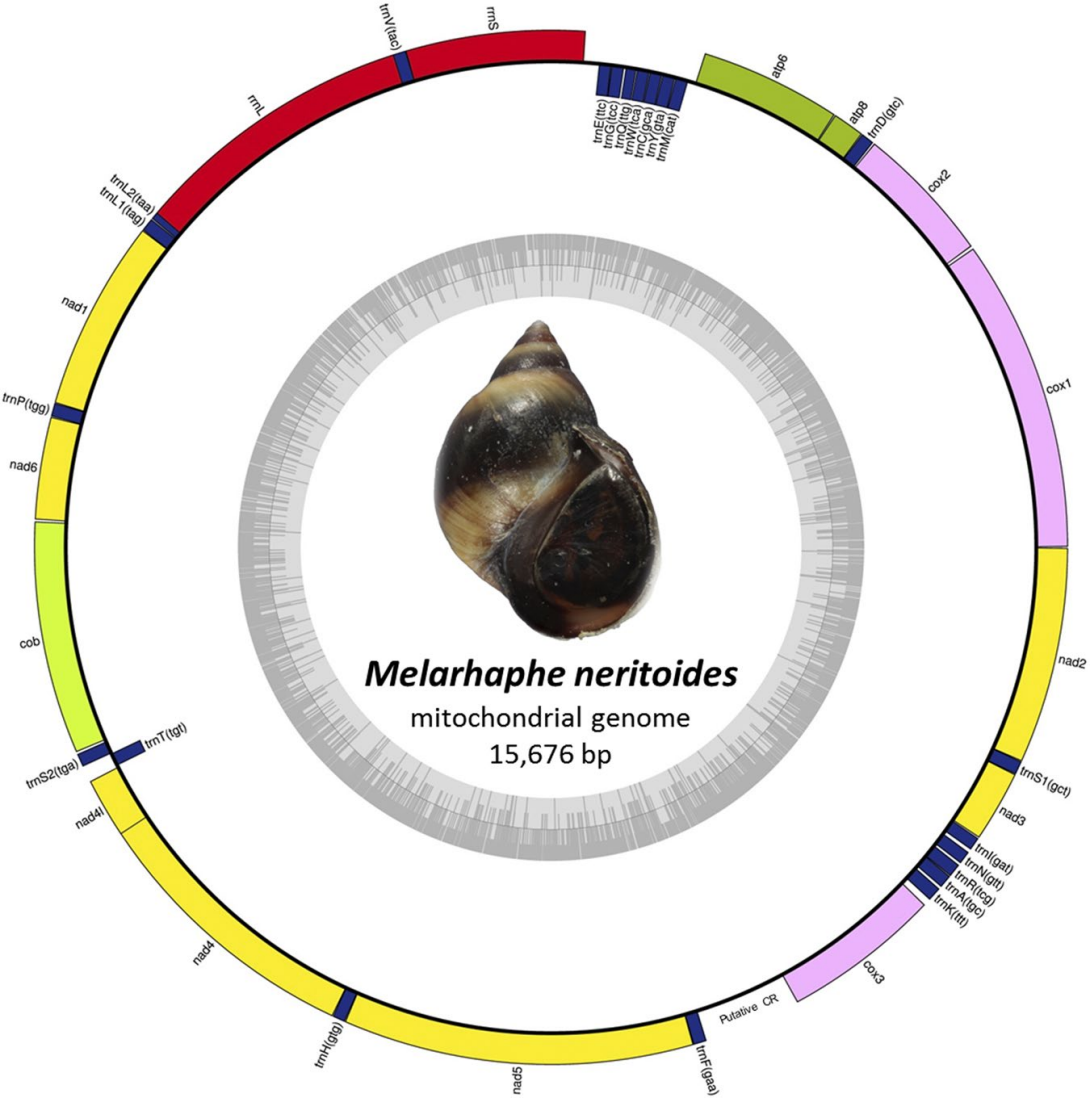
Gene map of the *Melarhaphe neritoides* mitogenome. Genes encoded on the plus strand are mapped outside the outer circle and are transcribed counterclockwise. Genes encoded on the minus strand are mapped inside the outer circle and are transcribed clockwise. The inner circle plot represents G + C% content; the darker the lines are, the higher their G + C% is. Photo credit: Yves Barette (RBINS).

The near complete mitogenome of *M. neritoides* is 15,676 bp long (GenBank accession number MH119311) and comprises 37 genes including 13 PCGs, 2 rRNAs genes, 22 tRNAs genes, and a putative non-coding Control Region (CR), typical for animal mitogenomes (Table 1, Fig. 1). The CR is partial (474 bp) and is flanked by *trnF(gaa)* and *cox3*. No repetitive sequences were detected in the CR. As in other molluscs, with the exception of some bivalves^21^, *M. neritoides* transcribes its mitogenome on two strands, with all genes, except eight tRNAs, being encoded on the plus strand. The plus strand in *M. neritoides* corresponds to the heavy strand, which is originally defined as the strand with the higher amount of G + T nucleotides, although often mistakenly assigned as the strand encoding the majority of genes^28^. There are five overlapping adjacent genes in *M. neritoides* [*rrnS* and *trnV(tac)*, *trnV(tac)* and *rrnL*, *rrnL* and *trnL2(taa)*, *nad4l* and *nad4*, *nad5* and *trnF(gaa)*] versus six in *Littorina* sp. [*trnG(tcc)* and *trnE(ttc)*, *rrnS and trnV(tac), trnV(tac)* and *rrnL*, *rrnL* and *trnL2(taa)*, *nad4l and nad4, nad3* and *trnS1(gct)*]. The *nad4l* and *nad4* genes in *M. neritoides* and *Littorina* sp. overlap over seven nucleotides, and over variable lengths (7–13 bp) in 60 out of the 64 other caenogastropods for which we had mitogenome data. Hence, this overlap seems a common feature to Caenogastropoda. In *M. neritoides* and *Littorina* sp. all PCGs start with the canonical ATG codon, except for *atp6* that starts with ATT in *M. neritoides*. The 13 stop codons are the two standard TAA and TAG stop codons, TAA being more frequent (77%) than TAG (23%), like *Littorina* sp. (62% TAA and 38% TAG). The pattern of association of these stop codons with their respective PCGs is identical between *M. neritoides* and *Littorina* sp., except for the stop codons in *atp6*, *atp8*, *cob*, *nad1*, *nad2*, *nad4*, *nad4l* and *nad6*. Non-coding intergenic sequences in *M. neritoides* (from 1 to 78 bp) and *Littorina* sp. (from 1 to 72 bp) have similar lengths, the longest being that between *trnE(ttc)* and *rrnS* in the four littorinid species. Yet, *M. neritoides* has fewer intergenic sequences than *Littorina* sp. (23 vs 28) and their total length is shorter (250 vs 328–335 bp).

**Table 1:** Organisation of the mitochondrial genome of *Melarhaphe neritoides* and comparison with *Littorina fabalis*, *Littorina obtusata* and *Littorina saxatilis*^25^.

Gene	Strand	Location	Start codon	Stop codon	Length (bp)	Intergenic nucleotides*	Amino acid changes**
*cox1*	+	1–1536	ATG	TAA	1536	11 **30**	10/511 (2%)
*cox2*	+	1548–2234	ATG	TAA	687	5 **2**	11/228 (5%)
*trnD(gtc)*	+	2240–2307			68 **69**	1	
*atp8*	+	2309–2467	ATG	TAA **TAG**	159	2 **13**	12/52 (23%)
*atp6*	+	2470–3165	ATT **ATG**	TAA **TAG**	696	38 **31**	35/231 (15%)
*trnM(cat)*	—	3204–3271			68	1	
*trnY(gta)*	—	3273–3340			68	1 **11**	
*trnC(gca)*	—	3342–3407			66 **65**	1	
*trnW(tca)*	—	3409–3474			66	2 **1**	
*trnQ(ttg)*	—	3477–3533			57 **58**	7 **11**	
*trnG(tcc)*	—	3541–3607			67	0 −**1**	
*trnE(ttc)*	—	3608–3672			65 **71**	78 **72**	
*rrnS*	+	3751–4632			882 **894**^a,b^ **895**^c^	−3	
*trnV(tac)*	+	4630–4696			67 **68**	−23 **−22**	
*rrnL*	+	4674–6087			1414 **1415**	−36 −**10**	
*trnL2(taa)*	+	6052–6121			70 **67**	2 **8**	
*trnL1(tag)*	+	6124–6191			68 **67**	0	
*nad1*	+	6192–7133	ATG	TAG **TAA**	942 **939**	0 **7**	42/313 (13%)
*trnP(tgg)*	+	7134–7200			67 **68**	1 **2**	
*nad6*	+	7202–7705	ATG	TAA **TAG**	504 **513**	8 **9**	61/167 (37%)
*cob*	+	7714–8853	ATG	TAG **TAA**	1140	9 **18**^a,b^ **17**^c^	38/379 (10%)
*trnS2(tga)*	+	8863–8929			67 **68**	0 **5**	
*trnT(tgt)*	—	8930–8999			70 **71**^a^	8	
*nad4l*	+	9008–9304	ATG	TAA **TAG**	297	−7	14/98 (14%)
*nad4*	+	9298–10668	ATG	TAA **TAG**^c^	1371	0 **8**^a^ **9**^b,c^	108/456 (24%)
*trnH(gtg)*	+	10669–10732			64 **66**	0 **1**	
*nad5*	+	10733–12454	ATG	TAA	1722 **1719**	−1 **21**^a,b^ **23**^c^	129/573 (23%)
*trnF(gaa)*	+	12454–12520			67 **69**	0	
CR (partial)		12521–12994			474	0	
*cox3*	+	12995–13774	ATG	TAA	780	32 **33**	19/259 (7%)
*trnK(ttt)*	+	13807–13878			72 **73**	11 **6**^a^ **5**^b^	
*trnA(tgc)*	+	13890–13957			68 **67**	1	
*trnR(tcg)*	+	13959–14027			69	10 **5**	
*trnN(gtt)*	+	14038–14104			67	15 **13**^a,c^ **14**^b^	
*trnI(gat)*	+	14120–14186			67 **69**	3 **4**	
*nad3*	+	14190–14543	ATG	TAA	354	0 **−1**	22/117 (19%)
*trnS1(gct)*	+	14544–14611			68 **67**	0	
*nad2*	+	14612–15673	ATG	TAG **TAA**	1062 **1059**	3 **5**	112/353 (32%)

Differences between *M. neritoides* and ^a^*Littorina fabalis*, ^b^*Littorina obtusata* and ^c^*Littorina saxatilis* are in bold. *Numbers of intergenic nucleotides separating a gene from the next one; negative values represent overlapping nucleotides in adjacent genes. **Number (and corresponding percentage) of residues in the amino acid sequence of *Melarhaphe neritoides* that differ with any of the three other species.

The overall nucleotide composition of the mitogenome of *M. neritoides* is AT-rich (66.3%), with A = 28.9%, C = 16.6%, G = 17.1% and T = 37.4% (Table 2). All regions of the mitogenome are AT-rich but with the lowest values in *rrnS* (48.6%) and *rrnL* (51.1%) and the highest values in *atp8* (69.2%). This AT content is in line with that of other caenogastropods (65.2–74.9%)^29^ except for the Vermetidae whose AT content is slightly lower (59–63%)^19^, and of gastropods (55–67%)^15^. There is a negative AT skew over the mitogenome (−0.129) indicating a significant bias towards the use of T over A, except in CR and the two rRNA genes in which A is more common. The positive GC skew over the mitogenome (+0.012) indicates a significant bias towards the use of G over C, except in *atp8*, *atp6*, *cob*, *nad4*, *nad5* and CR. As such, *M. neritoides* shows a conspicuous positive GC skew, like in many other Mollusca (+0.04)^7^, but in strong contrast with the negative GC skew (mean −0.13) in *Littorina* sp.

**Table 2:** Nucleotide composition of the mitochondrial genome of *Melarhaphe neritoides* and *Littorina* sp.

Region	A%	C%	G%	T%	A + T%	G + C%	AT skew	GC skew
whole mitogenome *M. neritoides*	28.9	16.6	17.1	37.4	66.3	33.7	−0.129	0.012
*cox1*	26.0	17.3	19.3	37.4	63.3	36.7	−0.180	0.055
*cox2*	29.1	17.5	18.5	34.9	64.0	36.0	−0.091	0.028
*atp8*	30.2	15.7	15.1	39.0	69.2	30.8	−0.127	−0.019
*atp6*	24.9	17.1	15.5	42.5	67.4	32.6	−0.261	−0.049
*rrnS*	30.7	13.8	37.5	17.9	48.6	51.3	0.263	0.462
*rrnL*	36.1	12.5	36.4	15.0	51.1	48.9	0.413	0.489
*nad1*	25.7	16.8	17.1	40.4	66.1	33.9	−0.222	0.009
*nad6*	26.8	15.7	16.1	41.5	68.3	31.8	−0.215	0.013
*cob*	24.6	18.7	17.7	39.0	63.6	36.4	−0.226	−0.027
*nad4l*	27.9	15.8	17.2	39.1	67.0	33.0	−0.167	0.042
*nad4*	26.4	19.0	15.2	39.4	65.8	34.2	−0.198	−0.111
*nad5*	25.7	20.1	15.9	38.3	64.0	36.0	−0.197	−0.117
CR (partial)	32.7	19.8	15.8	31.6	64.3	35.7	0.016	−0.112
*cox3*	24.4	18.6	22.2	34.9	59.3	40.8	−0.177	0.088
*nad3*	28.2	15.0	19.5	37.3	65.5	34.5	−0.139	0.130
*nad2*	27.7	13.2	17.5	41.6	69.3	30.7	−0.201	0.140
whole mitogenome *L. fabalis*	29.9	18.9	14.9	36.3	66.2	33.8	−0.097	−0.119
whole mitogenome *L. obtusata*	29.9	19.1	14.7	36.4	66.2	33.8	−0.098	−0.129
whole mitogenome *L. saxatilis*	30.4	18.9	14.1	36.5	66.9	33.1	−0.091	−0.145

### tRNA secondary structure

All 22 tRNA genes are present in the mitogenome of *M. neritoides*. They range in length from 57 to 72 bp. Only two tRNA genes do not fold into the typical cloverleaf secondary structure: *trnS2(uga)* has a dihydrouridine (DHU) arm (D-arm) that forms a loop without a stem (as in *Littorina* sp.), and *trnM(cau)* has a T-arm with a stem but without the loop, which is specific to *M. neritoides* (Fig. S1). It is unclear whether this unpaired *trnM* is functional. However, it is likely functional because truncated tRNAs lacking the D-arm or the T-arm in some nematodes and other metazoans are functional too^30^. The loss of the D-arm and/or T-arm in *trnS2* is an occasional event, that in Mollusca occurs far less frequently than in other metazoans^31^. Conversely, the D-arm in *trnS1(gct)* often lacks in metazoans, but is present in *M. neritoides*, *Littorina* sp., and most Mollusca^31^. Additionally, there are minor differences between *M. neritoides* and *L. saxatilis*, such as the cloverleaf structure of *trnR(tcg)*, *trnN(gtt)*, *trnH(gtg)*, *trnT(tgt)* and *trnV(tac)* that does not show a loop within the stem of the Acceptor arm, and that of *trnR(tcg)* and *trnL1(tag)* that does not show a loop within the stem of the T-arm (Fig. S1). These loops are also absent in the six species of Vermetidae for which mitogenomes are available^19^. There are fewer differences among the three closely related *Littorina* species, than between *Littorina* and *Melarhaphe*. In fact, the 22 cloverleaf structures of *L. fabalis* and *L. obtusata* are identical, and differ only from those of *L. saxatilis* by the absence of a loop in the Acceptor arm of *trnH(gtg)* and *trnV(tac)*.

Further comparative analyses at the population level among individuals of *M. neritoides* are needed to know whether the mitogenome features observed in one individual are representative for the species and for mtDNA hyperdiversity.

### Protein sequence evolution and selection

The proportion of amino acids differing between the 13 mtDNA-encoded proteins of *M. neritoides* and *Littorina* sp. varies from 2% in COX1 to 37% in NAD6 (Table 1). The most conserved protein sequences between *Melarhaphe* and *Littorina* are COX1 > COX2 > COX3 > COB, with ≤10% of amino acid differences, followed by NAD1 > NAD4L > ATP6 > NAD3 > ATP8, NAD5 > NAD4 >> NAD2 showing more than 10% amino acid differences, to the least conserved NAD6.

**Figure 2:**
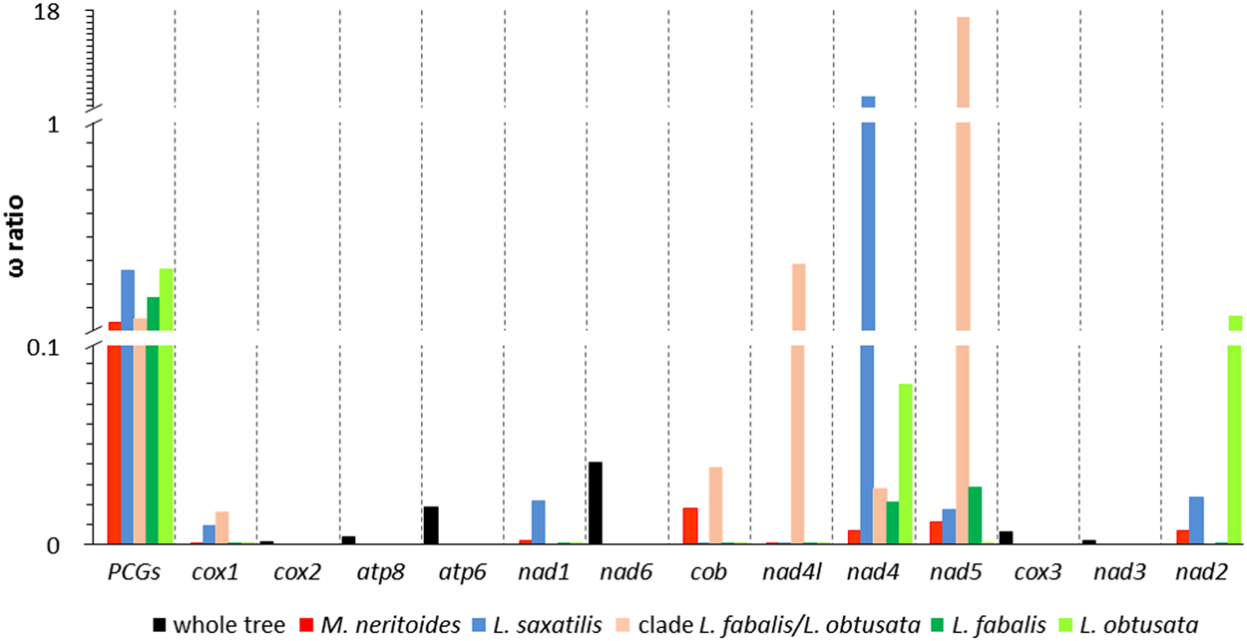
Evolutionary rates (ω) of amino acid substitutions for each protein-coding gene among four littorinid mitogenomes.

In both prokaryotes and eukaryotes, the rate of amino acid substitution is primarily determined by the protein expression level, which is in turn determined by the intensity of selection, while amino acid composition and the functional importance of a protein play a minor role in protein evolution^32^. Highly expressed proteins evolve slower because they are under stronger selective pressure, which strengthens mRNA folding, reduces protein mistranslation and misfolding, and avoids deleterious protein-protein misinteraction among protein surfaces^32^. The significant non-synonymous/synonymous substitution ratios (ω) for the concatenated PCGs reveal signatures of purifying (negative) selection (ω < 1) for all five branches of the tree topology used in the PAMLX analysis, i.e. *M. neritoides*, *L. saxatilis*, *L. fabalis*, *L. obtusata*, and the stem branch of *L. fabalis* + *L. obtusata* (Fig. 2, Supplementary Table S1). The ω values vary among branches, indicating stronger purifying selection in the mitogenome of *M. neritoides* (ω = 0.1361) and in the stem branch of *L. fabalis* + *L. obtusata* (ω = 0.1534), than in the mitogenome of *L. fabalis* (ω = 0.2438), *L. obtusata* (ω = 0.3667) and *L. saxatilis* (ω = 0.3595). Yet, analyses on single PCG suggest that selection acts differently among genes and branches, and show that although most PCGs are under purifying selection, a few of them are positively selected. On the one hand, most PCGs show very low ω values from 0.0001 to 0.0798 in all lineages, indicative of strong purifying selection, while *nad2* in *L. obtusata* (ω = 0.1638) and *nad4l* in the stem branch of *L. fabalis* + *L. obtusata* (ω = 0.3827) show ω values closer to 1, indicative of more relaxed purifying selection. In *M. neritoides*, genes under purifying selection are ranked as follows by decreasing strength of selection (increasing ω): *cox1*, *nad4l* > *cox2* > *nad1* > *nad3* > *atp8* > *cox3* > *nad2* > *nad4* > *nad5* > *cob* > *atp6* > *nad6*. This order differs from that in *Littorina* sp. and vertebrates^7^. Indeed, purifying selection in *cob* of *M. neritoides* is one order of magnitude weaker than in the other *co* genes (*cox1*, *cox2*, *cox3*), whereas in other metazoans purifying selection is usually of similar strength in the four *co* genes^7^. Nevertheless, the strength of purifying selection on *cob* in *M. neritoides* and the *Littorina* species lies in the range reported in other metazoans such as fishes^33^, insects and some vertebrates^7^. In contrast, *nad4* in *L. saxatilis* and *nad5* in the stem branch of *L. fabalis* + *L. obtusata* show significant (LRT >3.84) ω values above 1, which are exceptionally high (respectively ω = 3.5994 and ω = 16.8910) and higher than in other littorinid branches. This is indicative of strong positive selection on *nad4* and *nad5* in these two branches. Such positive selection has been reported in mitochondrial PCGs of other animals^34^, probably reflecting environmental adaptation (e.g. thermal adaption, hypoxia tolerance) or mitonuclear coadaptation^35^.

In conclusion, purifying selection seems a major evolutionary force acting on the mitogenomes of *M. neritoides*, *L. fabalis*, *L. obtusata* and *L. saxatilis*, and is expected to maintain crucial mitochondrial gene functions^7^, since mtDNA-encoded proteins are responsible for the oxidative phosphorylation. Yet, strong positive selection on *nad4* and *nad5* suggests that these genes play an important role in mitogenome evolution in *L. saxatilis* and in the stem branch of *L. fabalis* + *L. obtusata*. Positive selection on *nad4* in *L. saxatilis* might promote increasing adaptive mitogenome divergence between *L. saxatilis* and the other littorinids. Similarly, positive selection on *nad5* may promote adaptive divergence in *L. fabalis* and *L. obtusata*, and contribute to separate the clade *L. fabalis* + *L. obtusata* from the other littorinids.

Fourdrilis *et al*.^2^ detected positive selection in *M. neritoides* in *cox1* and *cob* using Fay & Wu’s H statistic that applies to all polymorphic nucleotide sites, hence including both synonymous and non-synonymous nucleotide sites. In the present study, no positive selection is detected in the 13 PCGs of *M. neritoides*, but purifying selection is detected on non-synonymous sites using the d_N_/d_S_ ratio. Therefore, positive selection acts on synonymous sites only, and purifying selection contributes to lowering rate of substitutions at non-synonymous sites in *M. neritoides*.

### Codon usage

**Figure 3:**
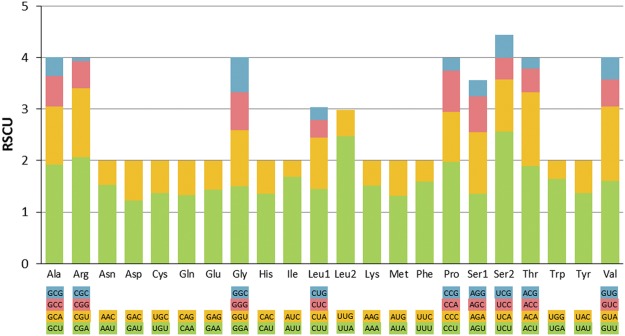
Relative synonymous codon usage (RSCU) of the mitochondrial genome of *Melarhaphe neritoides*. The 22 codon families consisting of a total of 62 two- and four-fold degenerate synonymous codons are plotted on the x-axis. The label for the 2 or 4 codons that compose each family is shown in the boxes below the x-axis, and the colours correspond to the colours in the stacked columns. The most used synonymous codon in each family is in green. The RSCU values are shown on the y-axis.

The usage of synonymous codons in PCGs is not random in *M. neritoides* as some codon families and codons are more frequently used (Fig. 3). The codon family encoding the amino acid Ser2 is the most prevalent of the 22 families, followed by the codon families encoding Ala, Arg, Gly, Pro, Thr and Val equally used among each other. Among the 62 synonymous codons (the two stop codons are excluded) of these 22 codon families, 29 codons are more frequently used (RSCU value >1), and account for less than half (46.77%) of the total set of codons (Supplementary Table S2). The five most frequently used (largest RSCU values) codons are Ser2 (UCU), Leu2 (UUA), Arg (CGA), Pro (CCU) and Ala (GCU) (RSCU = 2.57, 2.47, 2.07, 1.97, 1.92 respectively). They represent together 16.25% of the 3754 synonymous codons constituting the PCGs in *M. neritoides*. The least used codon is Arg (CGC) (RSCU = 0.07). This suggests selection of optimal synonymous codons for translational efficiency^36^, likely resulting from the strong purifying selection observed on non-synonymous nucleotide sites in the PCGs of *M. neritoides*.

There is an over-usage of two-fold and four-fold degenerate synonymous codons with A or T in the third position in comparison to other synonymous codons, and all 29 preferentially used codons end with A or T (U) (Supplementary Table S2). This suggests, in addition to the contribution of selection to codon usage bias, a role of higher mutation pressure at the third codon position from GC to AT than in the opposite direction (from AT to GC) and than at the first and second codon positions^36^.

The usage of the three codons UUC(F), AUG(M) and CAG(Q) significantly differs at the 5% level in *M. neritoides* from that in *Littorina* sp. (Supplementary Table S3). Even more codons show significant differences (UUC(F), CUC(L1), UUA(L2), AUC(I), AUG(M), CCC(P), UAC(Y), CAC(H), AAC(N), GAC(D), GAG(E) and GGC(G)) with *Naticarius hebraeus* (family Naticidae), which is expected with increasing phylogenetic divergence. However, no significant differences could be detected between the codon usage in *M. neritoides* and that in the other non-Latrogastropoda families Provannidae and Hydrobiidae, while in the even more distantly related species *Monoplex parthenopeus* (Ranellidae) belonging to Latrogastropoda two codons showed significantly different usage (AUG(M) and GGC(G)). There is therefore no clear evidence of an association between codon usage and phylogenetic relationship within Hypsogastropoda. After Bonferroni correction of the results above, none of the chi-square tests remains significant. Nevertheless, Bonferroni correction is designed to prevent any false positives, and hence may eliminate true positives too. However, after Bonferroni correction, the other littorinid *L. saxatilis* shows much more significant differences than *M. neritoides* with the same other species. Indeed, the usage of five codons significantly differs from that in *Naticarius hebraeus* (Naticidae), one codon in *Ifremeria nautilei* (Provannidae), one codon in *Potamopyrgus antipodarum* (Hydrobiidae), and one codon in *Monoplex parthenopeus* (Ranellidae) (Supplementary Table S3). Hence, mtDNA hyperdiversity in *M. neritoides* does not seem to be associated with greater or smaller biases in codon usage.

### Gene arrangement

**Figure 4:**
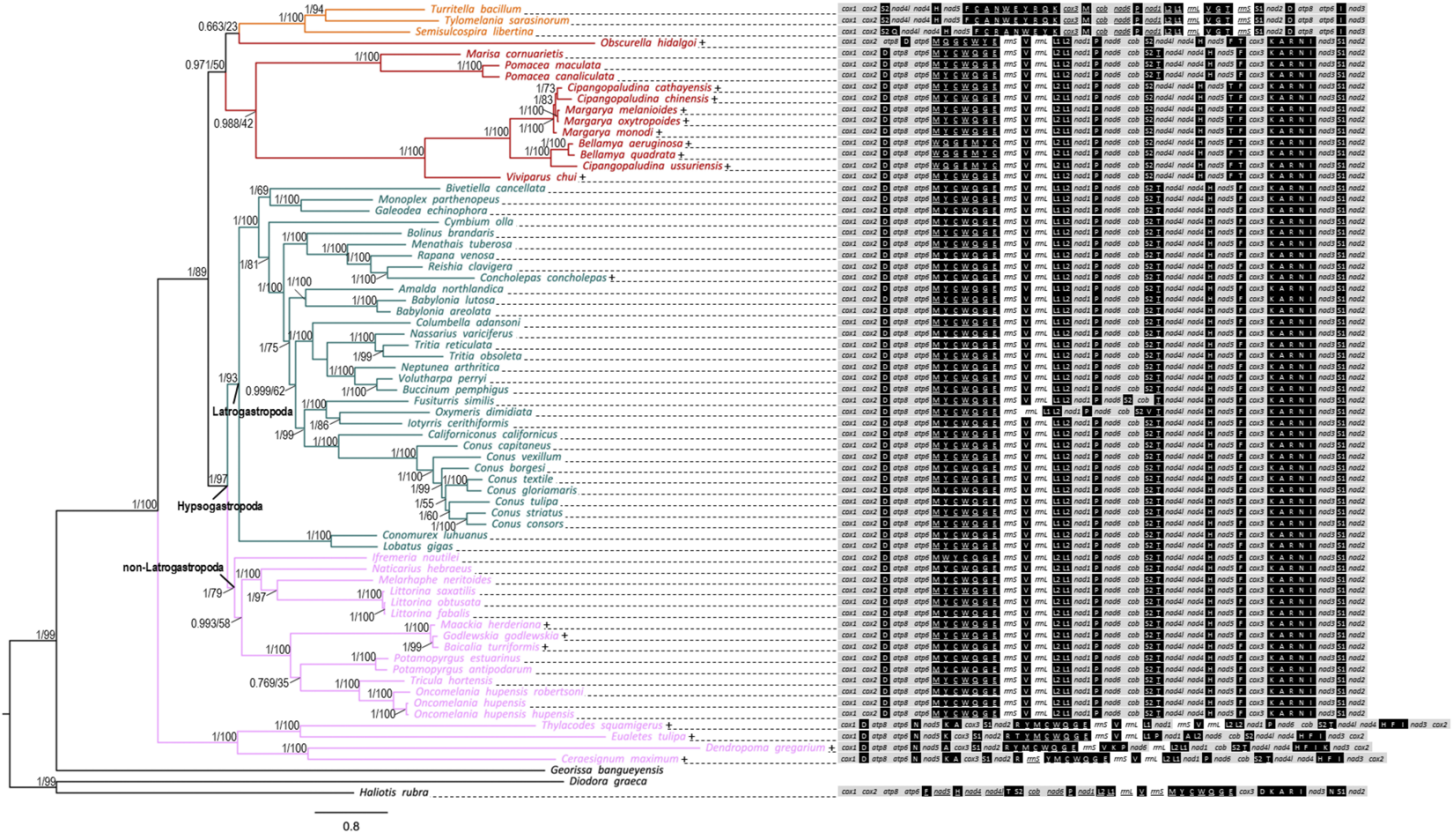
Gene order of the 13 protein-coding genes of *Melarhaphe neritoides* mitogenome drawn into a phylogenetic context including a total of 68 Caenogastropoda. Underlined genes are encoded on the minus strand. Symbol letters for tRNAs indicate the encoded amino acid and follows the IUPAC-IUB nomenclature for amino acids. Branches are colour-coded to represent the putative clades Architaenioglossa (red), Cerithiimorpha (orange), Latrogastropoda (green), non-Latrogastropoda Hypsogastropoda (pink), and outgroup taxa are left in black. Numbers at the nodes are Bayesian posterior probabilities (left) and ML bootstrap values (right). Branches with posterior probability >0.95 and bootstrap support value >70% are considered to be strongly supported. Scale bar is substitutions/site. Plus sign indicates long branch.

We focus here on the non-Latrogastropoda i.e. the hypsogastropods that are not included in Latrogastropoda (Fig. 4, Supplementary Table S4), to check for the phylogenetic context of *M. neritoides*, but see Osca *et al*.^26^ and the recently revised classification by Bouchet *et al*.^27^ for a more complete description of Caenogastropoda relationships. The non-Latrogastropoda corresponds largely to the former Littorinimorpha, which is a paraphyletic taxon^26,27^. The phylogeny of Caenogastropoda, based on the complete set of mitochondrial PCG sequence data of 68 caenogastropod species with most recently updated annotations, recovered six out of the seven putative families included within non-Latrogastropoda (Fig. 4). The 7^th^ putative family, i.e. Vermetidae, is placed as sister group to all other caenogastropods, probably due to long branch attraction or extensive gene rearrangements^19^. *Ifremeria nautilei* (Provannidae) is placed as sister group to non-Latrogastropoda, and shares the same tRNA gene order L2-L1, which supports its close relationship to non-Latrogastropoda^37^. The families Cassidae (*Galeodea echiniphora*), Ranellidae (*Monoplex parthenopeus*) and Strombidae (*Conomurex luhuanus*, *Lobatus gigas*) were affiliated to “Littorinimorpha” in the previous classifications, and are nested within Latrogastropoda in the current classification, which is consistent with their tRNAs in the same order L1-L2 as the most frequent gene order in Latrogastropoda. *Melarhaphe neritoides* is sister to the *Littorina* sp. with strong bootstrap support (95%) and maximal posterior probability (pp = 1). The gene order in *M. neritoides* is identical to the most frequent gene order of non-Latrogastropoda, including its closest relatives *Littorina* sp., hence mtDNA hyperdiversity does not seem to be associated with a particular gene order. This most frequent gene order is also found in one out of the 67 other caenogastropod taxa, the architaenioglossan *Marisa cornuarietis*, and is therefore not unique to non-Latrogastropoda.

Assuming that *Haliotis rubra* (subclass Vetigastropoda) represents the ancestral mtDNA gene order (with two additional derived changes) for gastropods^15^, the gene order showing the highest similarity to the ancestral gene order is that of either *Conus borgesi* (identical to *Cymbium olla*) or *Conomurex luhuanus* (identical to the 28 other Latrogastropoda and the two architaenioglossan *Pomacea canaliculata* and *P. maculata*) based on an evolutionary distance measure of similarity (398 common intervals). Yet, when relying on an evolutionary distance measure of dissimilarity, only *Conomurex luhuanus* shows a gene order closest to the ancestral one (11 breakpoints versus 12 in *Conus borgesi*). Moreover, the gene order in *Conomurex luhuanus* shows the fewest rearrangement events (five events vs. six in *Conus borgesi*) and thus better represents the ancestral gene order of Caenogastropoda. This means that, in contrast to the conclusion of Wang *et al*.^29^, *Conus borgesi* does not show the most conserved gene order. This is not surprising since, due to annotation correction in the present study, the gene order in *Conus borgesi* (and *Cymbium olla*) differs from that in *Conomurex luhuanus* on the direction of *trnT*, whereas it was identical in Wang *et al*.^29^ (who did not include *Conomurex luhuanus* itself in their study but did include 16 taxa with identical gene order to *Conomurex luhuanus*). Annotation quality has therefore influenced gene order analyses. The Caenogastropoda consensus gene order found in *Galeodea echiniphora* by Osca *et al*.^26^, which is identical to that of *Conomurex luhuanus*, is consistent with our conclusion, although the tRNAs L1 and L2 were not distinguished by the authors. However, the existence of gene rearrangement hot spots in mitogenomes^38^ suggests that this “ancestral” gene order represented by *Conomurex luhuanus* here may not be plesiomorphic (i.e. “conserved”), but rather reflects a homoplasic rearrangement.

Eighteen Caenogastropoda have significantly longer branches than the average root-to-tip distance (0.66) ranging from δ = +0.12 ± 0.03 to δ = +1.06 ± 0.08, indicating that these taxa have higher nucleotide substitution rates and evolve faster than others at the 1% level (CP value ≥99%) (Fig. 4). Among these long-branched caenogastropods, all Vermetidae show highly rearranged mitogenomes. The vermetid *Eualetes tulipa* even shows the most divergent gene order among Caenogastropoda (74 common intervals and 24 breakpoints), a result that again differs from the work by Wang *et al*.^29^, who identified the cerithiimorph *Turitella bacillum* as having the most divergent gene order (yet this species shows 84 common intervals, 22 breakpoints). Likewise, all Viviparidae, Megalomastomatidae and Baicaliidae are long-branched and show gene order rearrangements, which supports an association between high substitution rates and gene order rearrangement. In contrast, the only long-branched species within Latrogastropoda, *Concholepas concholepas*, shows the “ancestral” gene order, whereas the short-branched species *Conus borgesi*, *Cymbium olla* and *Fusiturris similis* do show mitogenome rearrangements, and while *Oxymeris dimidiata* shows mitogenome rearrangements but no significant difference in branch length. Furthermore, all short-branched taxa within non-Latrogastropoda show the most frequent non-Latrogastropoda gene order. Therefore, it seems that there is no clear association between high substitution rates and gene order rearrangement in Caenogastropoda. Stöger & Schrodl^21^ suggested a correlation between multiple rearrangements and increased substitution rates within Mollusca, which they observed in bivalves, patellogastropods and heterobranchs, but not in caenogastropods. This may be due to the sampling that was limited to 17 caenogastropods, none of which was long-branched (see their Supplementary Fig. 2). The present phylogeny suggests that an eventual association between mtDNA gene order rearrangements and increased substitution rates does not hold for all Caenogastropoda, but may apply to a few families and/or species only.

The significantly shorter branch length in *M. neritoides* than the average branch length in caenogastropods is indicative of a lower mtDNA substitution rate. Therefore, a high mtDNA mutation rate cannot be associated straightforwardly with a high mtDNA substitution rate. Under the neutral theory, the substitution rate is the substitution rate of neutral substitutions and is equal to *µ*^39^. In this context, a low substitution rate in *M. neritoides* may indicate that *µ* is high only in the hyperdiverse *cox1* and *cob* genes and is much lower in the other PCGs, which consequently lowers the global *µ* and substitution rate in the mitogenome. However in this work, we showed that substitutions at synonymous sites are under positive selection, and hence the substitution rate involves neutral and non-neutral substitutions. Under such non-neutrality, a low substitution rate in *M. neritoides* may indicate that a fraction from the large number of mutations that arise in *M. neritoides* is selected and fixed over time, and thus, all initial mutations do not contribute to the substitution rate.

### Mitogenome annotation methodology

In the absence of experimental data about the sequence and the length of mature mRNA transcripts, gene annotation is somewhat problematic. We followed a conservative methodology for annotating mitogenomes to limit the risk of spurious annotations. However, conservative methodology has two main inherent shortcomings. Firstly, by selecting the most frequent start codon in the mitogenome data set for the most consistent gene length among related species, peculiarities that truly occurred through evolution might be missed (e.g. shorter/longer gene lengths), particularly in variable PCGs such as *atp* and *nad* genes. And secondly, by inferring annotations in newly sequenced mitogenomes, based on a reference mitogenome instead of experimental data from the new mitogenomes, potential erroneous annotations in the reference mitogenome are spread to subsequent annotations. In this study, 41 out of 70 mitogenomes downloaded from GenBank were corrected following criteria detailed hereunder, urging the need of following a standardised protocol to annotate mitogenome data. Annotations were not updated in Genbank at our request, but can be updated at the request of the initial submitting authors.

## Conclusions

The relation between mtDNA hyperdiversity, mtDNA mutation rate and mitogenome evolution was investigated using the littorinid periwinkle *Melarhaphe neritoides*, in which mtDNA hyperdiversity is primarily caused by high mtDNA mutation rates. mtDNA hyperdiversity and high mutation rates do not seem to be associated with a particular mitogenome architecture, which is in line with the trend in Mollusca with the 37 mtDNA genes, the AT-rich nucleotide composition, the strand-specific distribution of genes, the most frequent use of ATG start codon and TAA stop codon, the negative AT skew (and hence positive GC skew), and gene order. Only transfer RNA *trnM* is atypical and unpaired, lacking the loop in the T-arm. mtDNA hyperdiversity is supposed to reflect neutral polymorphisms, yet, positive selection maintains or reduces the amount of synonymous polymorphism in *M. neritoides*, while strong purifying selection reduces the amount of non-synonymous polymorphism and is a major evolutionary force acting on the mitogenome of *M. neritoides*, as it does in the three other littorinids *Littorina fabalis*, *L. obtusata* and *L. saxatilis*. Purifying selection and mutational pressure contribute to codon usage bias, but the non-random usage of codons in *M. neritoides* is comparable to other littorinids, and hence, there is no obvious relation between mtDNA hyperdiversity or high mutation rates and codon usage. In a phylogenetic context, *M. neritoides* shows a low mtDNA substitution rate, suggesting that high mtDNA mutation rates are not necessarily associated with high mtDNA substitution rates. Synonymous substitutions in *M. neritoides* are not neutral, and hence substitution rate involves neutral and non-neutral substitutions and does not equal mutation rate. This may explain the lack of clear association between mtDNA hyperdiversity and high mutation rates on the one hand, and mitogenome architecture and evolution on the other. Yet, architaenioglossan and vermetid caenogastropods with high mtDNA substitution rates appear to show an association between their high substitution rate and gene order rearrangement.

## Methods

### Specimen collection and DNA extraction

We collected a specimen of *M. neritoides* on 6 July 2012 in the port of Varadouro, Faial island, Azores, Portugal (N 38.56633, W 28.77068), in accordance with regulations, and preserved it at −20 °C (frozen without ethanol) until DNA analysis. We extracted genomic DNA from foot muscle using the NucleoSpin® Tissue kit (Macherey-Nagel GmbH & Co. KG, Germany). All remaining body parts and the shell were deposited in the collections of the Royal Belgian Institute of Natural Sciences, Brussels (RBINS) under the general inventory number IG 32962 and specimen voucher INV.134051.

### Mitogenome sequencing and annotation

Library preparation with insert size of 250 bp, shotgun sequencing on an Illumina HiSeq. 4000 platform for 2.5 Gb of paired end reads, and *de novo* assembly of the mitogenome using SOAPdenovo-Trans (−K 71, −t 1), were performed by the Beijing Genomics Institute (Hong Kong) following manufacturer’s instructions and Tang *et al*.’s^40^ pipeline. The mitogenome was annotated with the MITOS WebServer^41^, followed by manual curation using Geneious 10.2.3^42^. We followed Boore^43^ for naming conventions, and Boore, *et al*.^44^ and Cameron^45^ for annotation recommendations. Despite these recommendations, we noted inconsistent annotations in 41 out of 70 mitogenomes downloaded from GenBank (December 2016) for the present study (see Supplementary Table S4). Revised annotations are provided in Supplementary Dataset 1. We corrected several start codon positions in *nad* genes which have very variable 5′ ends, following the methodology described hereunder. The quality of mitogenome data is influenced by, amongst others, the raw sequence quality, the sequence assembly and the annotation methods^46^. We therefore suggest that authors report the main criteria they used to annotate mitogenome data deposited in GenBank, as we do here. We manually corrected mitogenome annotations as follows: 1) PCGs were assumed to begin at the first eligible in-frame start codon in their 5′ end, i.e. the start codon nearest to the preceding gene without overlapping with it, and we ensured this start codon was suitable in terms of location and gene length by aligning the derived amino acid sequence with amino acid sequences of the same gene from closely related species; 2) Since both mtDNA strands are transcribed as polycistronic RNA, i.e. a single transcript which encodes more than one protein^47^, we considered it to be physically impossible to have gene overlap between two PCGs encoded on the same strand and in the same open reading frame, but possible if frames are different; 3) PCGs were assumed to end in the 3′ side at the first in-frame full stop codon, or at an abbreviated stop codon (TA- or T-- in invertebrates) ending immediately before the downstream tRNA in order to reduce overlap with downstream genes and to preserve gene length consistency among closely related species. Such an abbreviated codon results from the cleavage of the transcript at the 5′ and 3′ ends of tRNAs and tRNA-like secondary structures, and is subsequently completed with A by polyadenylation; 4) Duplicated genes were sorted out based on quality values provided in the MITOS analysis, and the inconsistent duplicate which shows low quality value was ruled out; 5) The boundaries of tRNA genes were those predicted by MITOS; 6) The boundaries of rRNA genes were those predicted by MITOS and were not assumed to extend to flanking genes, in order to avoid overestimating rRNA gene length; 7) The boundaries of predicted PCGs and rRNAs in *M. neritoides* were compared to the three mitogenomes of littorinids published to date, i.e. *Littorina fabalis*, *Littorina obtusata* and *Littorina saxatilis*^25^. The graphical representation of the *M. neritoides* mitogenome was drawn with OGDRAW^48^.

### Mitogenome composition and organization

We conducted analyses of nucleotide composition and relative synonymous codon usage (RSCU) using MEGA 7.0^49^. We investigated the relation between mtDNA hyperdiversity and RSCU, using a chi-square test and correcting for multiple test biases using the sequential Bonferroni procedure^50^, in order to know whether the bias in the RSCU of *M. neritoides* significantly differs from the bias in the RSCU of closely related species from the same family (i.e. three *Littorina* sp.) and from three different families (i.e. *Ifremeria nautilei*, *Naticarius hebraeus*, *Potamopyrgus antipodarum*) or of more distantly related species (i.e. the ranellid *Monoplex parthenopeus*) (Supplementary Table S3). For each pair of species, a total of 21 chi-square tests was performed (one test per codon family), in which the observed frequency of a codon is the count of this codon in *M. neritoides*, and the expected frequency of a codon is the count if this codon was equally used in the two species. We calculated nucleotide skew statistics using the formulas: AT skew = [A − T]/[A + T] and GC skew = [G − C]/[G + C]^51^. We predicted and compared secondary structures of tRNAs among the four littorinids using the MITOS WebServer. We used mitogenome sequences of 21 “Littorinimorpha” taxa and 46 other Caenogastropoda that were available in GenBank (see Supplementary Table S4), and mapped gene order of mitogenomes onto the phylogeny.

### Sequence divergence, protein sequence evolution and selection

We estimated protein sequence divergence in the 13 mtDNA-encoded proteins by calculating the proportion of amino acid residues that differ among *M. neritoides* and three *Littorina* species. We performed a maximum likelihood estimation of the ratio (ω) of non-synonymous (d_N_) to synonymous (d_S_) substitution rates^52^ to infer the direction and magnitude of natural selection acting on PCGs, using branch models which allow ω to vary among branches in the phylogeny^53,54^ as implemented in CODEML in the PAMLX 1.3.1 package^55^. The number of synonymous nucleotide substitutions per synonymous site, d_S_, is largely determined by mutation rate only, whereas the number of non-synonymous nucleotide substitutions per non-synonymous site, d_N_, is determined jointly by mutation rate and selection. Therefore, ω is determined by selection only. We compared two branch models, viz. the free-ratios model which assumes one ω ratio for each branch in the tree, and the two-ratios model which assumes one ω ratio for the foreground branch (user-specified *a priori*, one lineage at a time) putatively under positive selection and one ω ratio for the remaining background branches, to the null model which yields an averaged ω_0_ for the whole tree. Significance was assessed by a likelihood ratio test.

### Caenogastropoda phylogeny and mitogenome rearrangement

We employed Bayesian (BI) and Maximum Likelihood (ML) approaches, implemented respectively in MrBayes 3.2.6^56^ and RAxML 8.2.10^57^, respectively, both hosted on the CIPRES Science Gateway^58^, to carry out the phylomitogenomic analysis of 68 Caenogastropoda taxa (see Supplementary Table S4) based on their concatenated PCGs. Neritimorpha and Vetigastropoda were used as outgroup (see Supplementary Table S4). Although Heterobranchia has been recovered as sister group to Caenogastropoda^59–61^, it was not chosen as outgroup because it induces long branch attraction artefacts in mitogenome-based phylogenies^21^. In absence of Heterobranchia, Neritimorpha is the expected closest living sister group to Caenogastropoda, while Vetigastropoda is sister to the group comprising Neritimorpha and Caenogastropoda^62,63^, hence the tree was rooted on Vetigastropoda. Single PCG nucleotide sequences were translated into amino acid sequences before aligning to avoid spurious gaps within codons, and translated back to nucleotide sequences, using Geneious. The concatenated nucleotide dataset was divided into 39 data blocks, for the first, second and third codon positions of the 13 PCGs. The optimal partition strategy of each block (Supplementary Table S5), restricted to GTR + G model of sequence evolution as recommended by Stamatakis^64^, was selected by PartitionFinder 2.1.1^65,66^. The final BI consensus tree was computed from the combination of two independent MCMC runs of 60,000,000 generations each, sampling every 100 generations and discarding the first 15,000,000 generations. Convergence was assessed in TRACER. 1.6^67^. The bootstrap ML consensus tree was inferred from 1000 replicates. Long branches, i.e. branches significantly longer than the average branch length across the tree, were diagnosed by applying the Branch Length Test (BLT) using LINTREE (http://www.personal.psu.edu/nxm2/software.htm)^68^. LINTREE produced a neighbour-joining tree based on the Tamura-Nei + G model of sequence evolution, the most similar model to GTR + G found in LINTREE, and estimated for each taxon the difference delta (δ) between the average branch length across the tree and the root-to-tip distance of the taxon. Events of mitogenome rearrangement were determined using CREx^69^ and we compared gene orders found in Caenogastropoda to the ancestral gastropod gene order in *Haliotis rubra*. The higher the number of common intervals and the lowest number of breakpoints between pair of taxa, the more similar are the gene orders.

## Electronic supplementary material

Supplementary information

Supplementary Table S3

Supplementary Dataset 1

## Data Availability

The mitogenome sequence of *Melarhaphe neritoides* is available in NCBI GenBank database under the accession number MH119311. The revised mitogenome annotations are provided in the form of *.gb* files (Supplementary Dataset 1).
