# Supplementary material for: Relation between mitochondrial DNA hyperdiversity, mutation rate and mitochondrial genome evolution in Melarhaphe neritoides (Gastropoda: Littorinidae) and other Caenogastropoda
Source: Sci Rep. 2018 Dec 19;8:17964. doi: 10.1038/s41598-018-36428-7 (PMC6299273; doi:10.1038/s41598-018-36428-7)
Supplement: Supplementary file 1 — Supplementary information [file 41598_2018_36428_MOESM1_ESM.pdf]

Scientific Reports

SUPPLEMENTARY INFORMATION

Relation between mitochondrial DNA hyperdiversity, mutation rate and mitochondrial genome evolution in *Melarhaphe neritoides* (Gastropoda: Littorinidae) and other Caenogastropoda

Séverine Fourdrilis[1,*], Antonio M. de Frias Martins[2], Thierry Backeljau[1,3]

[1] *Royal Belgian Institute of Natural Sciences, Rue Vautier 29, B-1000 Brussels, Belgium,*

[2] *CIBIO, Centro de Investigação em Biodiversidade e Recursos Genéticos, InBIO Laboratório Associado, Pólo dos Açores, Departamento de Biologia da Universidade dos Açores, Campus de Ponta Delgada, Apartado 1422, 9501-801 Ponta Delgada, Açores, Portugal,*

[3] *Evolutionary Ecology Group, University of Antwerp, Universiteitplein 1, B-2610 Antwerp, Belgium*

* Corresponding author: Séverine Fourdrilis severine.fourdrilis@gmail.com

Supplementary Table S1. Results from the PAMLX analyses. Models selected to best fit the data and best describe natural selection acting on protein-coding genes (PCGS) in *Melarhaphe neritoides*, *Littorina fabalis*, *Littorina obtusata* and *Littorina saxatilis*. Values shaded in grey indicate positive selection.

**Region**	PCGs	*cox1*	*cox2*	*atp8*	*atp6*	*nad1*	*nad6*	*cob*	*nad4l*	*nad4*	*nad5*	*cox3*	*nad3*	*nad2*
**ML tree**	A	A	A	A	A	B	A	A	A	A	A	A	A	B
**Model**	1	1	0	0	0		0	1	1	1	1	0	0	1
**LRT**	34.75	5.55				3.87		4.14	6.91	7.17	11.10			8.56
$\boldsymbol{\omega_0}$			0.0018	0.0041	0.0185		0.0408					0.0062	0.0021	
$\boldsymbol{\omega_{L.\ fabalis}}$	0.2438	0.0001				0.0001		0.0001	0.0001	0.0212	0.0287			0.0001
$\boldsymbol{\omega_{L.\ obtusata}}$	0.3667	0.0001				0.0001		0.0001	0.0001	0.0798	0.0001			0.1638
$\boldsymbol{\omega_{clade\ fabalis/obtusata}}$	0.1534	0.0164						0.0386	0.3827	0.0278	16.8910			
$\boldsymbol{\omega_{M.\ neritoides}}$	0.1361	0.0010				0.0019		0.0184	0.0010	0.0072	0.0115			0.0068
$\boldsymbol{\omega_{L.\ saxatilis}}$	0.3595	0.0096				0.0218		0.0001	0.0001	3.5994	0.0177			0.0238

ML tree topology: A = ((*Littorina fabalis*,*Littorina obtusata*),*Melarhaphe neritoides*,*Littorina saxatilis*); B = (*Littorina fabalis*,*Littorina obtusata*,*Melarhaphe neritoides*,*Littorina saxatilis*).

Model: 0 = null model, 1 = branch model 1 (free-ratios model); the branch model 2 (two-ratios model) was never selected.

LRT: value of the likelihood ratio test for the comparison of the free-ratios model against the null model, significantly greater than the critical chi-square value for 1 degree of freedom with a significance level of 0.05 (χ^2 = 3.84).

ω: d_N/d_S ratio for the null model (ω_0) or the free-ratios model for each branch in the tree.

Supplementary Table S2. Relative synonymous codon usage (RSCU) of each amino acid in the mitochondrial genomes of *Littorina fabalis*, *Littorina obtusata*, *Littorina saxatilis* and *Melarhaphe neritoides*. More frequently used codons (RSCU > 1) are in bold.

Melarhaphe neritoides									
**Amino acid**	**Codon**	**Count**	**RSCU**	**%**	**Amino acid**	**Codon**	**Count**	**RSCU**	**%**
Ala	**GCA(A)**	**77**	**1.13**	**2.05**	Lys	**AAA(K)**	**68**	**1.51**	**1.81**
Ala	GCC(A)	40	0.59	1.07	Lys	AAG(K)	22	0.49	0.59
Ala	GCG(A)	25	0.37	0.67	Met	**AUA(M)**	**123**	**1.31**	**3.28**
Ala	**GCU(A)**	**131**	**1.92**	**3.49**	Met	AUG(M)	65	0.69	1.73
Arg	**CGA(R)**	**31**	**2.07**	**0.83**	Phe	UUC(F)	66	0.41	1.76
Arg	CGC(R)	1	0.07	0.03	Phe	**UUU(F)**	**253**	**1.59**	**6.74**
Arg	CGG(R)	8	0.53	0.21	Pro	CCA(P)	29	0.81	0.77
Arg	**CGU(R)**	**20**	**1.33**	**0.53**	Pro	CCC(P)	35	0.97	0.93
Asn	AAC(N)	31	0.48	0.83	Pro	CCG(P)	9	0.25	0.24
Asn	**AAU(N)**	**97**	**1.52**	**2.58**	Pro	**CCU(P)**	**71**	**1.97**	**1.89**
Asp	GAC(D)	30	0.78	0.80	Ser1	**AGA(S1)**	**58**	**1.19**	**1.55**
Asp	**GAU(D)**	**47**	**1.22**	**1.25**	Ser1	AGC(S1)	34	0.70	0.91
Cys	UGC(C)	12	0.63	0.32	Ser1	AGG(S1)	15	0.31	0.40
Cys	**UGU(C)**	**26**	**1.37**	**0.69**	Ser1	**AGU(S1)**	**66**	**1.36**	**1.76**
Gln	**CAA(Q)**	**50**	**1.33**	**1.33**	Ser2	**UCA(S2)**	**49**	**1.01**	**1.31**
Gln	CAG(Q)	25	0.67	0.67	Ser2	UCC(S2)	20	0.41	0.53
Glu	**GAA(E)**	**64**	**1.44**	**1.70**	Ser2	UCG(S2)	22	0.45	0.59
Glu	GAG(E)	25	0.56	0.67	Ser2	**UCU(S)**	**125**	**2.57**	**3.33**
Gly	**GGA(G)**	**95**	**1.50**	**2.53**	Thr	**ACA(T)**	**59**	**1.42**	**1.57**
Gly	GGC(G)	43	0.68	1.15	Thr	ACC(T)	19	0.46	0.51
Gly	GGG(G)	47	0.74	1.25	Thr	ACG(T)	9	0.22	0.24
Gly	**GGU(G)**	**69**	**1.09**	**1.84**	Thr	**ACU(T)**	**79**	**1.90**	**2.10**
His	CAC(H)	26	0.64	0.69	Trp	**UGA(W)**	**90**	**1.65**	**2.40**
His	**CAU(H)**	**55**	**1.36**	**1.47**	Trp	UGG(W)	19	0.35	0.51
Ile	AUC(I)	42	0.31	1.12	Tyr	UAC(Y)	45	0.63	1.20
Ile	**AUU(I)**	**227**	**1.69**	**6.05**	Tyr	**UAU(Y)**	**98**	**1.37**	**2.61**
Leu1	CUA(L)	99	0.99	2.64	Val	**GUA(V)**	**90**	**1.45**	**2.40**
Leu1	CUC(L)	35	0.35	0.93	Val	GUC(V)	32	0.52	0.85
Leu1	CUG(L)	24	0.24	0.64	Val	GUG(V)	27	0.44	0.72
Leu1	**CUU(L)**	**145**	**1.45**	**3.86**	Val	**GUU(V)**	**99**	**1.60**	**2.64**
Leu2	**UUA(L)**	**248**	**2.47**	**6.61**	stop codon	UAA(*)	9	1.50	0.24
Leu2	UUG(L)	51	0.51	1.36	stop codon	UAG(*)	3	0.50	0.08
					Total		*3754*	*64*	*100*

Note: % = Count/3754*100.

Littorina fabalis									
**Amino acid**	**Codon**	**Count**	**RSCU**	**%**	**Amino acid**	**Codon**	**Count**	**RSCU**	**%**
Ala	GCA(A)	62	0.94	1.65	Lys	**AAA(K)**	**85**	**1.81**	**2.26**
Ala	**GCC(A)**	**67**	**1.02**	**1.78**	Lys	AAG(K)	9	0.19	0.24
Ala	GCG(A)	5	0.08	0.13	Met	**AUA(M)**	**160**	**1.68**	**4.26**
Ala	**GCU(A)**	**129**	**1.96**	**3.44**	Met	AUG(M)	30	0.32	0.80
Arg	**CGA(R)**	**32**	**2.13**	**0.85**	Phe	UUC(F)	103	0.64	2.74
Arg	CGC(R)	10	0.67	0.27	Phe	**UUU(F)**	**220**	**1.36**	**5.86**
Arg	CGG(R)	3	0.20	0.08	Pro	CCA(P)	37	0.98	0.99
Arg	**CGU(R)**	**15**	**1.00**	**0.40**	Pro	CCC(P)	24	0.64	0.64
Asn	AAC(N)	44	0.69	1.17	Pro	CCG(P)	5	0.13	0.13
Asn	**AAU(N)**	**83**	**1.31**	**2.21**	Pro	CCU(P)	85	2.25	2.26
Asp	**GAC(D)**	**44**	**1.19**	**1.17**	Ser1	**AGA(S1)**	**54**	**1.16**	**1.44**
Asp	GAU(D)	30	0.81	0.80	Ser1	AGC(S1)	36	0.77	0.96
Cys	**UGC(C)**	**22**	**1.19**	**0.59**	Ser1	AGG(S1)	7	0.15	0.19
Cys	UGU(C)	15	0.81	0.40	Ser1	AGU(S1)	44	0.94	1.17
Gln	**CAA(Q)**	**71**	**1.78**	**1.89**	Ser2	**UCA(S2)**	**68**	**1.45**	**1.81**
Gln	CAG(Q)	9	0.23	0.24	Ser2	**UCC(S2)**	**63**	**1.35**	**1.68**
Glu	**GAA(E)**	**80**	**1.80**	**2.13**	Ser2	UCG(S2)	6	0.13	0.16
Glu	GAG(E)	9	0.20	0.24	Ser2	**UCU(S2)**	**96**	**2.05**	**2.56**
Gly	**GGA(G)**	**82**	**1.33**	**2.18**	Thr	**ACA(T)**	**57**	**1.31**	**1.52**
Gly	GGC(G)	48	0.78	1.28	Thr	ACC(T)	35	0.80	0.93
Gly	GGG(G)	31	0.50	0.83	Thr	ACG(T)	7	0.16	0.19
Gly	**GGU(G)**	**86**	**1.39**	**2.29**	Thr	**ACU(T)**	**75**	**1.72**	**2.00**
His	CAC(H)	36	0.88	0.96	Trp	**UGA(W)**	**88**	**1.57**	**2.34**
His	**CAU(H)**	**46**	**1.12**	**1.23**	Trp	UGG(W)	24	0.43	0.64
Ile	AUC(I)	53	0.37	1.41	Tyr	UAC(Y)	48	0.66	1.28
Ile	**AUU(I)**	**235**	**1.63**	**6.26**	Tyr	**UAU(Y)**	**97**	**1.34**	**2.58**
Leu1	**CUA(L1)**	**104**	**1.05**	**2.77**	Val	**GUA(V)**	**85**	**1.44**	**2.26**
Leu1	CUC(L1)	65	0.65	1.73	Val	GUC(V)	36	0.61	0.96
Leu1	CUG(L1)	12	0.12	0.32	Val	GUG(V)	8	0.14	0.21
Leu1	**CUU(L1)**	**143**	**1.44**	**3.81**	Val	**GUU(V)**	**107**	**1.81**	**2.85**
Leu2	**UUA(L2)**	**245**	**2.47**	**6.53**	stop codon	UAA(*)	9	1.50	0.24
Leu2	UUG(L2)	27	0.27	0.72	stop codon	UAG(*)	3	0.50	0.08
					Total		*3754*	*64*	*100*

Note: % = Count/3754*100.

Littorina obtusata									
**Amino acid**	**Codon**	**Count**	**RSCU**	**%**	**Amino acid**	**Codon**	**Count**	**RSCU**	**%**
Ala	GCA(A)	62	0.95	1.65	Lys	**AAA(K)**	**85**	**1.81**	**2.27**
Ala	GCC(A)	61	0.93	1.63	Lys	AAG(K)	9	0.19	0.24
Ala	GCG(A)	4	0.06	0.11	Met	**AUA(M)**	**161**	**1.70**	**4.30**
Ala	**GCU(A)**	**135**	**2.06**	**3.60**	Met	AUG(M)	28	0.30	0.75
Arg	**CGA(R)**	**33**	**2.20**	**0.88**	Phe	UUC(F)	102	0.63	2.72
Arg	CGC(R)	10	0.67	0.27	Phe	**UUU(F)**	**220**	**1.37**	**5.87**
Arg	CGG(R)	2	0.13	0.05	Pro	**CCA(P)**	**38**	**1.01**	**1.01**
Arg	**CGU(R)**	**15**	**1.00**	**0.40**	Pro	CCC(P)	25	0.66	0.67
Asn	AAC(N)	46	0.73	1.23	Pro	CCG(P)	4	0.11	0.11
Asn	**AAU(N)**	**80**	**1.27**	**2.14**	Pro	**CCU(P)**	**84**	**2.23**	**2.24**
Asp	**GAC(D)**	**43**	**1.16**	**1.15**	Ser1	**AGA(S1)**	**55**	**1.17**	**1.47**
Asp	GAU(D)	31	0.84	0.83	Ser1	AGC(S1)	37	0.79	0.99
Cys	**UGC(C)**	**22**	**1.19**	**0.59**	Ser1	AGG(S1)	6	0.13	0.16
Cys	UGU(C)	15	0.81	0.40	Ser1	AGU(S1)	44	0.94	1.17
Gln	**CAA(Q)**	**71**	**1.78**	**1.89**	Ser2	**UCA(S2)**	**68**	**1.45**	**1.81**
Gln	CAG(Q)	9	0.23	0.24	Ser2	**UCC(S2)**	**63**	**1.34**	**1.68**
Glu	**GAA(E)**	**79**	**1.78**	**2.11**	Ser2	UCG(S2)	5	0.11	0.13
Glu	GAG(E)	10	0.22	0.27	Ser2	**UCU(S2)**	**97**	**2.07**	**2.59**
Gly	**GGA(G)**	**83**	**1.37**	**2.22**	Thr	**ACA(T)**	**59**	**1.34**	**1.57**
Gly	GGC(G)	47	0.78	1.25	Thr	ACC(T)	37	0.84	0.99
Gly	GGG(G)	29	0.48	0.77	Thr	ACG(T)	6	0.14	0.16
Gly	**GGU(G)**	**83**	**1.37**	**2.22**	Thr	**ACU(T)**	**74**	**1.68**	**1.97**
His	CAC(H)	37	0.90	0.99	Trp	**UGA(W)**	**92**	**1.66**	**2.46**
His	**CAU(H)**	**45**	**1.10**	**1.20**	Trp	UGG(W)	19	0.34	0.51
Ile	AUC(I)	54	0.38	1.44	Tyr	UAC(Y)	47	0.64	1.25
Ile	**AUU(I)**	**232**	**1.62**	**6.19**	Tyr	**UAU(Y)**	**99**	**1.36**	**2.64**
Leu1	**CUA(L1)**	**104**	**1.05**	**2.78**	Val	**GUA(V)**	**85**	**1.43**	**2.27**
Leu1	CUC(L1)	64	0.64	1.71	Val	GUC(V)	37	0.62	0.99
Leu1	CUG(L1)	9	0.09	0.24	Val	GUG(V)	7	0.12	0.19
Leu1	**CUU(L1)**	**144**	**1.45**	**3.84**	Val	**GUU(V)**	**108**	**1.82**	**2.88**
Leu2	**UUA(L2)**	**244**	**2.46**	**6.51**	stop codon	UAA(*)	9	1.50	0.24
Leu2	UUG(L2)	31	0.31	0.83	stop codon	UAG(*)	3	0.50	0.08
					Total		*3747*	*64*	*100*

Note: % = Count/3747*100.

Littorina saxatilis									
**Amino acid**	**Codon**	**Count**	**RSCU**	**%**	**Amino acid**	**Codon**	**Count**	**RSCU**	**%**
Ala	GCA(A)	64	0.98	1.70	Lys	**AAA(K)**	**82**	**1.74**	**2.18**
Ala	GCC(A)	60	0.92	1.60	Lys	AAG(K)	12	0.26	0.32
Ala	GCG(A)	8	0.12	0.21	Met	**AUA(M)**	**158**	**1.69**	**4.21**
Ala	**GCU(A)**	**130**	**1.98**	**3.46**	Met	AUG(M)	29	0.31	0.77
Arg	**CGA(R)**	**33**	**2.20**	**0.88**	Phe	UUC(F)	101	0.63	2.69
Arg	CGC(R)	9	0.60	0.24	Phe	**UUU(F)**	**222**	**1.37**	**5.91**
Arg	CGG(R)	2	0.13	0.05	Pro	**CCA(P)**	**41**	**1.08**	**1.09**
Arg	**CGU(R)**	**16**	**1.07**	**0.43**	Pro	CCC(P)	20	0.53	0.53
Asn	AAC(N)	45	0.71	1.20	Pro	CCG(P)	4	0.11	0.11
Asn	**AAU(N)**	**82**	**1.29**	**2.18**	Pro	**CCU(P)**	**87**	**2.29**	**2.32**
Asp	**GAC(D)**	**39**	**1.08**	**1.04**	Ser1	**AGA(S1)**	**50**	**1.07**	**1.33**
Asp	GAU(D)	33	0.92	0.88	Ser1	AGC(S1)	39	0.83	1.04
Cys	**UGC(C)**	**22**	**1.22**	**0.59**	Ser1	AGG(S1)	8	0.17	0.21
Cys	UGU(C)	14	0.78	0.37	Ser1	AGU(S1)	44	0.94	1.17
Gln	**CAA(Q)**	**73**	**1.78**	**1.94**	Ser2	**UCA(S2)**	**63**	**1.34**	**1.68**
Gln	CAG(Q)	9	0.22	0.24	Ser2	**UCC(S2)**	**62**	**1.32**	**1.65**
Glu	**GAA(E)**	**80**	**1.80**	**2.13**	Ser2	UCG(S2)	10	0.21	0.27
Glu	GAG(E)	9	0.20	0.24	Ser2	**UCU(S2)**	**99**	**2.11**	**2.64**
Gly	**GGA(G)**	**84**	**1.36**	**2.24**	Thr	**ACA(T)**	**59**	**1.34**	**1.57**
Gly	GGC(G)	55	0.89	1.47	Thr	ACC(T)	38	0.86	1.01
Gly	GGG(G)	27	0.44	0.72	Thr	ACG(T)	6	0.14	0.16
Gly	**GGU(G)**	**81**	**1.31**	**2.16**	Thr	**ACU(T)**	**73**	**1.66**	**1.94**
His	CAC(H)	35	0.86	0.93	Trp	**UGA(W)**	**96**	**1.71**	**2.56**
His	**CAU(H)**	**46**	**1.14**	**1.23**	Trp	UGG(W)	16	0.29	0.43
Ile	AUC(I)	62	0.43	1.65	Tyr	UAC(Y)	45	0.62	1.20
Ile	**AUU(I)**	**228**	**1.57**	**6.07**	Tyr	**UAU(Y)**	**100**	**1.38**	**2.66**
Leu1	**CUA(L1)**	**108**	**1.09**	**2.88**	Val	**GUA(V)**	**88**	**1.49**	**2.34**
Leu1	CUC(L1)	60	0.60	1.60	Val	GUC(V)	34	0.58	0.91
Leu1	CUG(L1)	10	0.10	0.27	Val	GUG(V)	6	0.10	0.16
Leu1	**CUU(L1)**	**151**	**1.52**	**4.02**	Val	**GUU(V)**	**108**	**1.83**	**2.88**
Leu2	**UUA(L2)**	**243**	**2.45**	**6.47**	stop codon	UAA(*)	8	1.33	0.04
Leu2	UUG(L2)	24	0.24	0.64	stop codon	UAG(*)	4	0.67	0.02
					Total		*3754*	*64*	*100*

Note: % = Count/3754*100.

Supplementary Table S4. Summary of sample information from the Caenogastropoda, Neritimorpha and Vetigastropoda subclasses used in the present study. Classification from Bouchet *et al.* (2017) [1].

Taxon	Superfamily	Family	Species	Accession Number	Reference
**Caenogastropoda**					
Grade Architaenioglossa	Ampullarioidea	Ampullariidae	*Marisa cornuarietis*	NC_025334	[2]
			Pomacea canaliculata	NC_024586 *	[3]
			Pomacea maculata	NC_027503 *	[4]
	Viviparoidea	Viviparidae	*Bellamya aeruginosa*	NC_035735 *	[5]
			Bellamya quadrata	NC_031850 *	[5]
			Cipangopaludina cathayensis	NC_025577 *	[6]
			Cipangopaludina chinensis	NC_035734 *	[5]
			Cipangopaludina ussuriensis	NC_035754	[5]
			Margarya melanioides	NC_035587 *	[5]
			Margarya monodi	NC_035585 *	[5]
			Margarya oxytropoides [a]	NC_035586 *	[5]
			Viviparus chui	NC_035733 *	[5]
	Cyclophoroidea	Megalomastomatidae	*Obscurella hidalgoi* [b]	NC_028004 *	[7]
Subcohort Hypsogastropoda	Abyssochrysoidea	Provannidae	*Ifremeria nautilei*	NC_024642	[8]
	Littorinoidea	Littorinidae	*Littorina fabalis*	KU952092	[9]
			Littorina obtusata	KU952093	[9]
			Littorina saxatilis	KU952094	[9]
			Melarhaphe neritoides	MH119311	this study
	Naticoidea	Naticidae	*Naticarius hebraeus*	NC_028002 *	[7]
	Rissooidea	Baicaliidae	*Baicalia turriformis*	NC_035869 *	unpublished

			Godlewskia godlewskia	NC_035870 *	unpublished
			Maackia herderiana	NC_035871 *	unpublished
	Truncatelloidea	Hydrobiidae	*Potamopyrgus antipodarum*	NC_020790 *	[10]
			Potamopyrgus estuarinus	NC_021595 *	[10]
		Pomatiopsidae	*Oncomelania hupensis*	NC_013073 *	[11]
			Oncomelania hupensis hupensis	NC_012899 *	[11]
			Oncomelania hupensis robertsoni	NC_013187 *	[11]
			Tricula hortensis	NC_013833 *	[11]
	Vermetoidea	Vermetidae	*Ceraesignum maximum* [c]	NC_014583 *	[12]
			Dendropoma gregarium	NC_014580 *	[12]
			Eualetes tulipa	NC_014585	[12]
			Thylacodes squamigerus	NC_014588	[12]
Superorder Latrogastropoda	Stromboidea	Strombidae	*Conomurex luhuanus* [d]	NC_035726 *	[13]
			Lobatus gigas [e]	NC_024932	[14]
	Tonnoidea	Cassidae	*Galeodea echiniphora*	NC_028003	[7]
		Ranellidae	*Monoplex parthenopeus* [f]	NC_013247 *	[15]
Order Neogastropoda	Buccinoidea	Buccinidae	*Buccinum pemphigus*	NC_029373	unpublished
			Neptunea arthritica	KU246047 *	[16]
			Volutharpa perryi	NC_028183	unpublished
		Columbellidae	*Columbella adansoni*	KP716637	[7]
		Nassariidae	*Tritia obsoleta* [g]	NC_007781 *	[17]
			Tritia reticulata [h]	NC_013248 *	[15]
			Nassarius variciferus [i]	NC_029173 *	unpublished
	Conoidea	Clavatulidae	*Fusiturris similis*	NC_013242 *	[15]
		Conidae	*Californiconus californicus*	NC_032377	[18]
			Conus borgesi	NC_013243 *	[15]

			Conus capitaneus	NC_030354 *	unpublished
			Conus consors	NC_023460	[19]
			Conus gloriamaris	NC_030213	unpublished
			Conus striatus	NC_030536	unpublished
			Conus textile	NC_008797 *	unpublished
			Conus tulipa	NC_027518	[20]
			Conus vexillum	NC_035007	unpublished
		Terebridae	*Oxymeris dimidiata* [j]	NC_013239 *	[15]
		Turridae	*Iotyrris cerithiformis* [k]	NC_008098	[21]
	Muricoidea	Muricidae	*Bolinus brandaris*	NC_013250 *	[15]
			Concholepas concholepas	NC_017886 *	[22]
			Menathais tuberosa	NC_031405	[23]
			Rapana venosa	NC_011193	[24]
			Reishia clavigera [l]	NC_010090 *	[25]
	Olivoidea	Ancillariidae	*Amalda northlandica*	NC_014403	[26]
	unassigned superfamliy	Babyloniidae	*Babylonia areolata*	NC_023080 *	unpublished
			Babylonia lutosa	NC_028628	[27]
	Volutoidea	Cancellariidae	*Bivetiella cancellata* [m]	NC_013241 *	[15]
		Volutidae	*Cymbium olla*	NC_013245 *	[15]
Subcohort Cerithiimorpha	Cerithioidea	Turritellidae	*Turritella bacillum*	NC_029717 *	[28]
		Pachychilidae	*Tylomelania sarasinorum*	NC_030263	[29]
		Semisulcospiridae	*Semisulcospira libertina*	NC_023364	[30]
**Neritimorpha (outgroup)**					
Order Cycloneritida	Hydrocenoidea	Hydrocenidae	*Georissa bangueyensis*	KU342664	[31]
**Vetigastropoda (outgroup)**					
Order Lepetellida	Fissurelloidea	Fissurellidae	*Diodora graeca*	KT207825	[32]

	Haliotoidea	Haliotidae	*Haliotis rubra*	NC_005940	[33]

[a - m] Names in the original publication were respectively: *Cipangopaludina dianchiensis, Cochlostoma hidalgoi, Dendropoma maximum, Strombus luhuanus, Strombus gigas, Cymatium parthenopeum, Ilyanassa obsolete, Nassarius reticulatus, Varicinassa variciferus, Terebra dimidiata, Xenuroturris cerithiformis, Thais clavigera* and *Cancellaria cancellata*. These species names have been updated in GenBank on the 18th of October, 2018.

* Genbank entry originally containing incorrect annotation(s) and subsequently corrected for the purpose of the present study.

Supplementary Table S5. The best partitioning scheme selected by PartitionFinder for the BI and ML phylogenetic analyses.

Subset	Best model	# sites	Regions
1	GTR+G	521	cox1_pos1
2	GTR+G	520	cox1_pos2
3	GTR+G	520	cox1_pos3
4	GTR+G	239	cox2_pos1
5	GTR+G	239	cox2_pos2
6	GTR+G	238	cox2_pos3
7	GTR+G	424	atp8_pos1, nad3_pos1, nad6_pos1
8	GTR+G	114	atp8_pos2
9	GTR+G	114	atp8_pos3
10	GTR+G	256	atp6_pos1
11	GTR+G	924	nad4_pos2, nad6_pos2, atp6_pos2
12	GTR+G	255	atp6_pos3
13	GTR+G	344	nad1_pos1
14	GTR+G	445	nad4l_pos2, nad1_pos2
15	GTR+G	343	nad1_pos3
16	GTR+G	188	nad6_pos3
17	GTR+G	386	cob_pos1
18	GTR+G	892	cob_pos2, cox3_pos2
19	GTR+G	385	cob_pos3
20	GTR+G	101	nad4l_pos1
21	GTR+G	101	nad4l_pos3
22	GTR+G	480	nad4_pos1
23	GTR+G	479	nad4_pos3
24	GTR+G	603	nad5_pos1
25	GTR+G	603	nad5_pos2
26	GTR+G	602	nad5_pos3
27	GTR+G	506	cox3_pos1
28	GTR+G	505	cox3_pos3
29	GTR+G	120	nad3_pos2
30	GTR+G	119	nad3_pos3
31	GTR+G	412	nad2_pos1
32	GTR+G	412	nad2_pos2
33	GTR+G	411	nad2_pos3

Supplementary Figure S1. Putative cloverleaf structures of tRNA genes in the mitogenome of (a) *Melarhaphe neritoides*, (b) *Littorina fabalis*, (c) *Littorina obtusata* and (d) *Littorina saxatilis*. Grey boxes mark regions showing differences between *Melarhaphe neritoides* and *Littorina* sp..

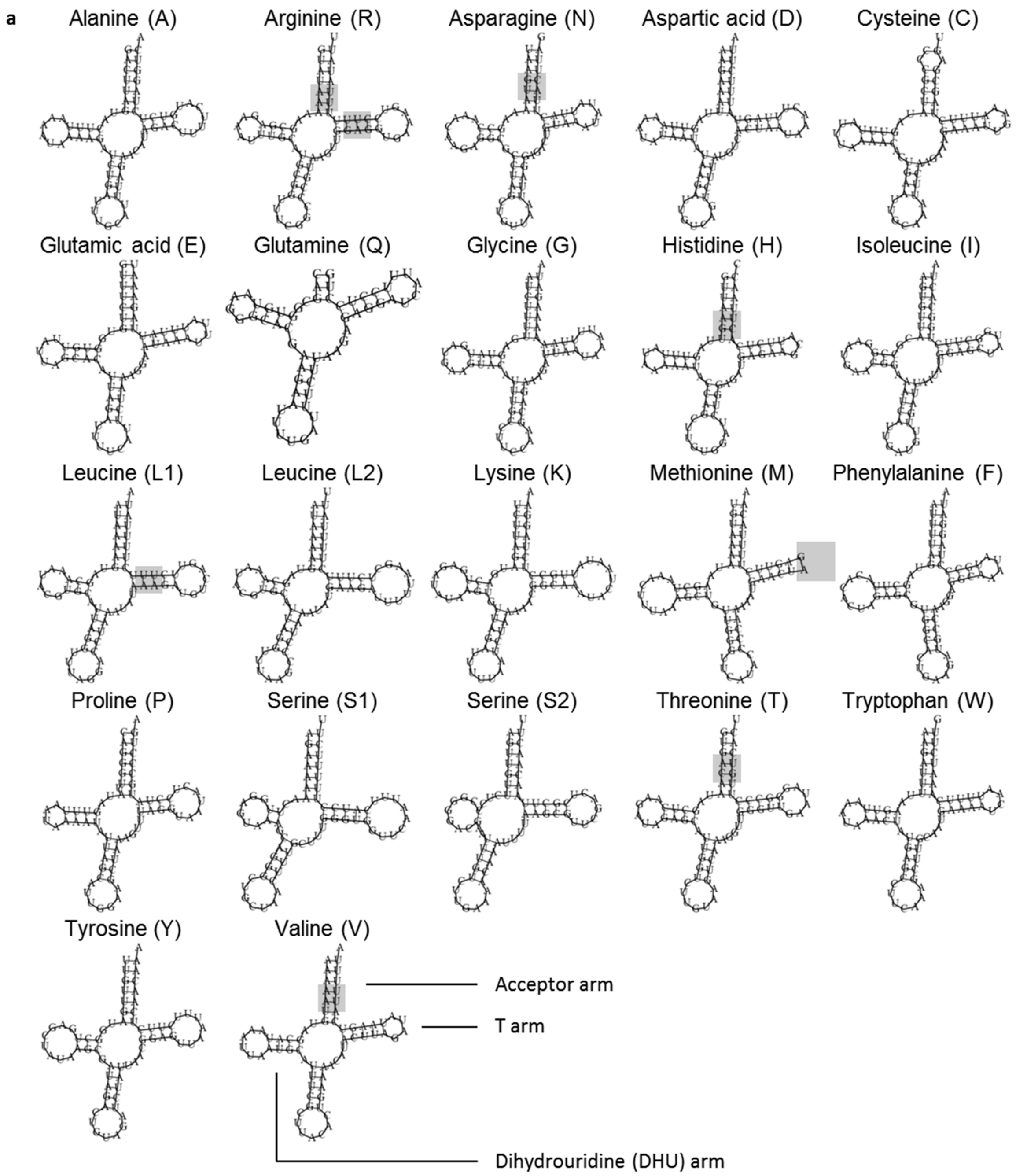

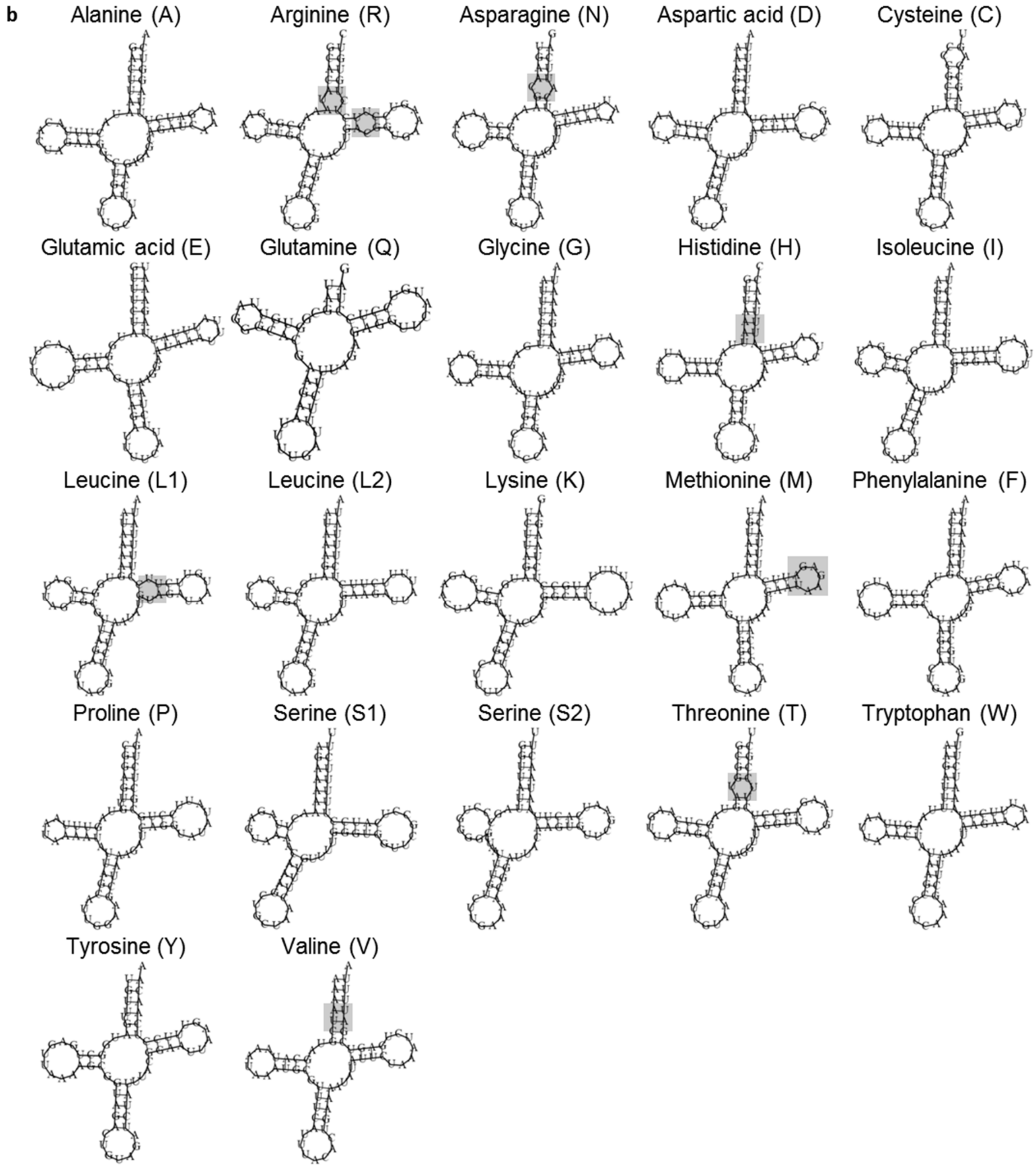
b
Alanine (A)
Arginine (R)
Asparagine (N)
Aspartic acid (D)
Cysteine (C)
Glutamic acid (E)
Glutamine (Q)
Glycine (G)
Histidine (H)
Isoleucine (I)
Leucine (L1)
Leucine (L2)
Lysine (K)
Methionine (M)
Phenylalanine (F)
Proline (P)
Serine (S1)
Serine (S2)
Threonine (T)
Tryptophan (W)
Tyrosine (Y)
Valine (V)

c

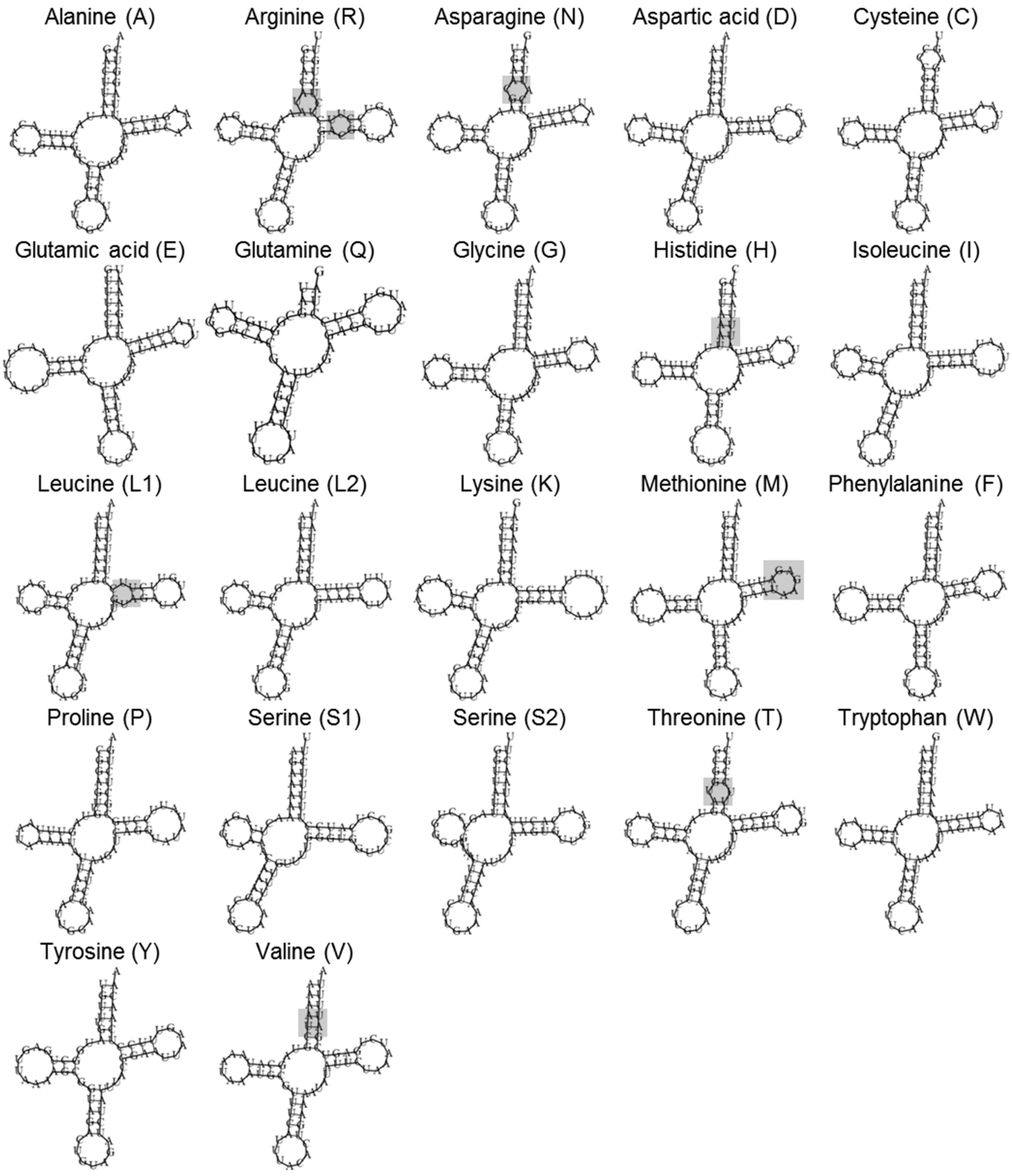
Alanine (A)
Arginine (R)
Asparagine (N)
Aspartic acid (D)
Cysteine (C)
Glutamic acid (E)
Glutamine (Q)
Glycine (G)
Histidine (H)
Isoleucine (I)
Leucine (L1)
Leucine (L2)
Lysine (K)
Methionine (M)
Phenylalanine (F)
Proline (P)
Serine (S1)
Serine (S2)
Threonine (T)
Tryptophan (W)
Tyrosine (Y)
Valine (V)

d

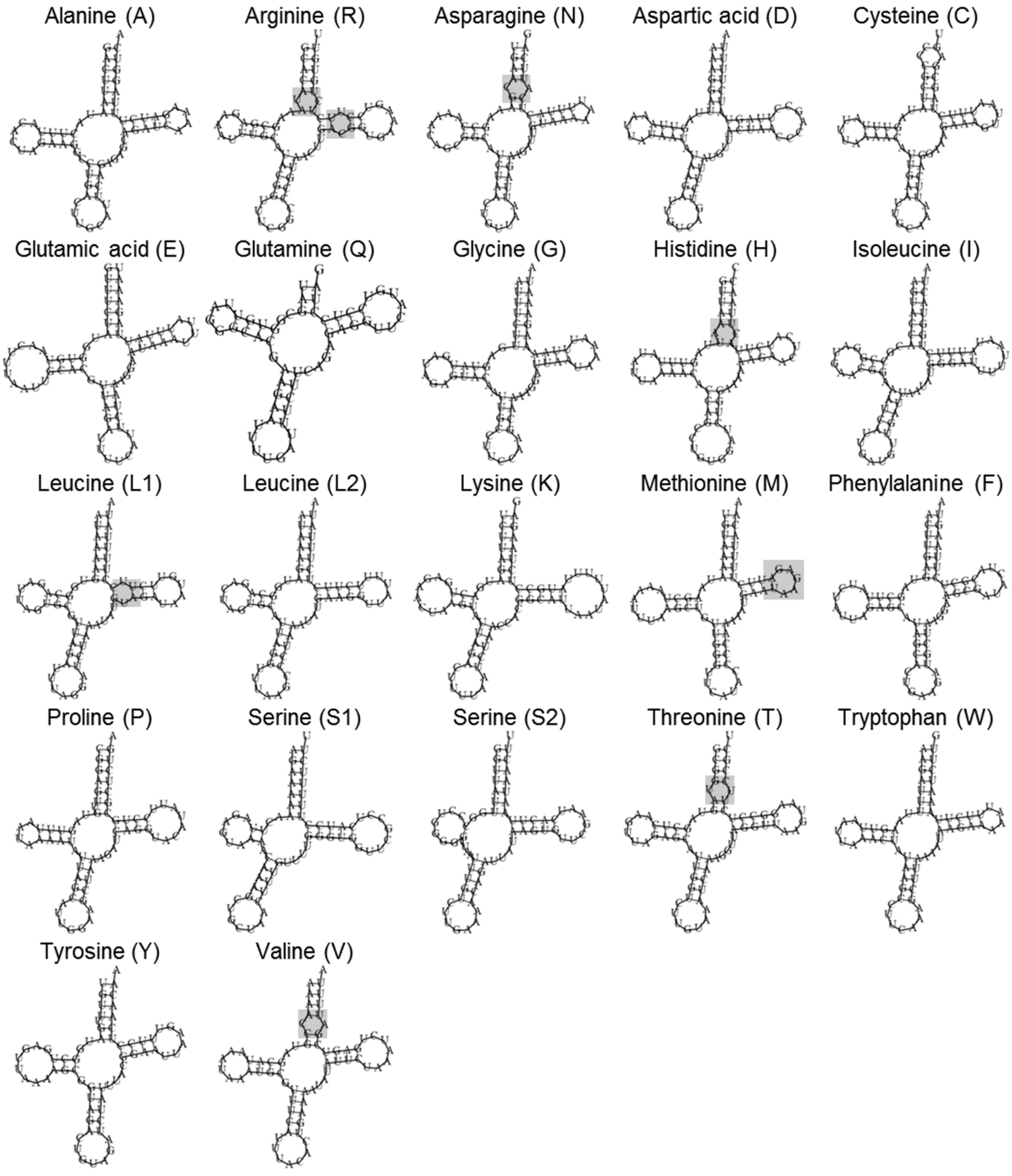
Alanine (A)
Arginine (R)
Asparagine (N)
Aspartic acid (D)
Cysteine (C)
Glutamic acid (E)
Glutamine (Q)
Glycine (G)
Histidine (H)
Isoleucine (I)
Leucine (L1)
Leucine (L2)
Lysine (K)
Methionine (M)
Phenylalanine (F)
Proline (P)
Serine (S1)
Serine (S2)
Threonine (T)
Tryptophan (W)
Tyrosine (Y)
Valine (V)

1 Bouchet, P. *et al.* Revised classification, nomenclator and typification of Gastropod and Monoplacophoran families. *Malacologia* **61**, 1-526, doi:10.4002/040.061.0201 (2017).

2 Wang, M. & Qiu, J.-W. Complete mitochondrial genome of the giant ramshorn snail *Marisa cornuarietis* (Gastropoda: Ampullariidae). *Mitochondrial DNA Part A* **27**, 1734-1735, doi:10.3109/19401736.2014.961145 (2016).

3 Zhou, X. *et al.* The complete mitochondrial genome of *Pomacea canaliculata* (Gastropoda: Ampullariidae). *Mitochondrial DNA Part A* **27**, 884-885, doi:10.3109/19401736.2014.919488 (2014).

4 Yang, Q. *et al.* The mitochondrial genome of *Pomacea maculata* (Gastropoda: Ampullariidae). *Mitochondrial DNA Part A* **27**, 2895-2896, doi:10.3109/19401736.2015.1060426 (2015).

5 Wang, J.-G., Zhang, D., Jakovlić, I. & Wang, W.-M. Sequencing of the complete mitochondrial genomes of eight freshwater snail species exposes pervasive paraphyly within the Viviparidae family (Caenogastropoda). *PLoS ONE* **12**, e0181699, doi:10.1371/journal.pone.0181699 (2017).

6 Yang, H. *et al.* The complete mitochondrial genome of the mudsnail *Cipangopaludina cathayensis* (Gastropoda: Viviparidae). *Mitochondrial DNA Part A* **27**, 1892-1894, doi:10.3109/19401736.2014.971274 (2014).

7 Osca, D., Templado, J. & Zardoya, R. Caenogastropod mitogenomics. *Mol. Phylogen. Evol.* **93**, 118-128, doi:http://dx.doi.org/10.1016/j.ympev.2015.07.011 (2015).

8 Osca, D., Templado, J. & Zardoya, R. The mitochondrial genome of *Ifremeria nautilei* and the phylogenetic position of the enigmatic deep-sea Abyssochrysoidea (Mollusca: Gastropoda). *Gene* **547**, 257-266, doi:10.1016/j.gene.2014.06.040 (2014).

9 Marques, J. P. *et al.* Comparative mitogenomic analysis of three species of periwinkles: *Littorina fabalis*, *L. obtusata* and *L. saxatilis*. *Marine Genomics* **32**, 41-47, doi:10.1016/j.margen.2016.10.006 (2017).

10 Neiman, M., Hehman, G., Miller, J. T., Logsdon, J. M. & Taylor, D. R. Accelerated mutation accumulation in asexual lineages of a freshwater snail. *Mol. Biol. Evol.* **27**, 954-963, doi:10.1093/molbev/msp300 (2010).

11 Zhao, Q.-P., Zhang, S. H., Deng, Z.-R., Jiang, M.-S. & Nie, P. Conservation and variation in mitochondrial genomes of gastropods *Oncomelania hupensis* and *Tricula hortensis*, intermediate host snails of *Schistosoma* in China. *Mol. Phylogen. Evol.* **57**, 215-226, doi:https://doi.org/10.1016/j.ympev.2010.05.026 (2010).

12 Rawlings, T. A., MacInnis, M. J., Bieler, R., Boore, J. L. & Collins, T. M. Sessile snails, dynamic genomes: gene rearrangements within the mitochondrial genome of a family of caenogastropod molluscs. *BMC Genomics* **11**, 440, doi:10.1186/1471-2164-11-440 (2010).

13 Zhao, Z.-y., Tu, Z.-g., Bai, L.-r. & Cui, J. Characterization of an endangered marine strombid gastropod *Strombus luhuanus* complete mitochondrial genome. *Conservation Genetics Resources*, doi:10.1007/s12686-017-0764-7 (2017).

14 Márquez, E. J., Castro, E. R. & Alzate, J. F. Mitochondrial genome of the endangered marine gastropod *Strombus gigas* Linnaeus, 1758 (Mollusca: Gastropoda). *Mitochondrial DNA Part A* **27**, 1516-1517, doi:10.3109/19401736.2014.953118 (2016).

15 Cunha, R. L., Grande, C. & Zardoya, R. Neogastropod phylogenetic relationships based on entire mitochondrial genomes. *BMC Evol. Biol.* **9**, 210, doi:10.1186/1471-2148-9-210 (2009).

16 Hao, Z. L. *et al.* The complete mitochondrial genome of *Neptunea arthritica cumingii* Crosse, (Gastropoda: Buccinidae). *Mitochondrial DNA Part B* **1**, 220-221, doi:10.1080/23802359.2016.1155421 (2016).

17 Simison, W. B., Lindberg, D. R. & Boore, J. L. Rolling circle amplification of metazoan mitochondrial genomes. *Mol. Phylogen. Evol.* **39**, 562-567, doi:https://doi.org/10.1016/j.ympev.2005.11.006 (2006).

18 Uribe, J. E., Puillandre, N. & Zardoya, R. Beyond Conus: Phylogenetic relationships of Conidae based on complete mitochondrial genomes. *Mol. Phylogen. Evol.* **107**, 142-151, doi:https://doi.org/10.1016/j.ympev.2016.10.008 (2017).

19 Brauer, A. *et al.* The mitochondrial genome of the venomous cone snail *Conus consors*. *PLoS ONE* **7**, e51528, doi:10.1371/journal.pone.0051528 (2012).

20 Chen, P.-W. *et al.* The complete mitochondrial genome of *Conus tulipa* (Neogastropoda: Conidae). *Mitochondrial DNA Part A* **27**, 2738-2739, doi:10.3109/19401736.2015.1046172 (2016).

21 Bandyopadhyay, P. K., Stevenson, B. J., Cady, M. T., Olivera, B. M. & Wolstenholme, D. R. Complete mitochondrial DNA sequence of a Conoidean gastropod, *Lophiotoma* (*Xenuroturris*) *cerithiformis*: Gene order and gastropod phylogeny. *Toxicon* **48**, 29-43, doi:https://doi.org/10.1016/j.toxicon.2006.04.013 (2006).

22 Núñez-Acuña, G., Aguilar-Espinoza, A. & Gallardo-Escárate, C. Complete mitochondrial genome of *Concholepas concholepas* inferred by 454 pyrosequencing and mtDNA expression in two mollusc populations. *Comparative Biochemistry and Physiology Part D: Genomics and Proteomics* **8**, 17-23, doi:https://doi.org/10.1016/j.cbd.2012.10.004 (2013).

23 Sung, J.-M., Karagozlu, M. Z., Lee, J., Kwak, W. & Kim, C.-B. The complete mitochondrial genome of *Menathais tuberosa* (Gastropoda, Neogastropoda, Muricidae) collected from Chuuk Lagoon. *Mitochondrial DNA Part B* **1**, 468-469, doi:10.1080/23802359.2016.1186516 (2016).

24 Sun, X. & Yang, A. The complete mitochondrial genome of *Rapana venosa* (Gastropoda, Muricidae). *Mitochondrial DNA Part A* **27**, 1471-1472, doi:10.3109/19401736.2014.953097 (2014).

25 Ki, J.-S. *et al.* Mitochondrial genome of *Thais clavigera* (Mollusca: Gastropoda): Affirmation of the conserved, ancestral gene pattern within the mollusks. *Mol. Phylogen. Evol.* **54**, 1016-1020, doi:https://doi.org/10.1016/j.ympev.2009.12.003 (2010).

26 McComish, B. J., Hills, S. F. K., Biggs, P. J. & Penny, D. Index-free de novo assembly and deconvolution of mixed mitochondrial genomes. *Genome Biology and Evolution* **2**, 410-424, doi:10.1093/gbe/evq029 (2010).

27 Xiong, G. *et al.* The complete mitochondrial genome of the *Babylonia lutosa*. *Mitochondrial DNA* **26**, 187-188, doi:10.3109/19401736.2013.873935 (2015).

28 Zeng, L., Wang, Y., Zhang, J. & Wu, C. Complete mitochondrial genome of *Turritella terebra bacillum*. *Mitochondrial DNA Part B* **1**, 350-351, doi:10.1080/23802359.2016.1144088 (2016).

29 Hilgers, L., Grau, J. H., Pfaender, J. & von Rintelen, T. The complete mitochondrial genome of the viviparous freshwater snail *Tylomelania sarasinorum* (Caenogastropoda: Cerithioidea). *Mitochondrial DNA Part B* **1**, 330-331, doi:10.1080/23802359.2016.1172046 (2016).

30 Zeng, T., Yin, W., Xia, R., Fu, C. & Jin, B. Complete mitochondrial genome of a freshwater snail, *Semisulcospira libertina* (Cerithioidea: Semisulcospiridae). *Mitochondrial DNA* **26**, 897-898, doi:10.3109/19401736.2013.861449 (2015).

31 Uribe, J. E., Colgan, D., Castro, L. R., Kano, Y. & Zardoya, R. Phylogenetic relationships among superfamilies of Neritimorpha (Mollusca: Gastropoda). *Mol. Phylogen. Evol.* **104**, 21-31, doi:10.1016/j.ympev.2016.07.021 (2016).

32 Uribe, J. E., Kano, Y., Templado, J. & Zardoya, R. Mitogenomics of Vetigastropoda: insights into the evolution of pallial symmetry. *Zoologica Scripta* **45**, 145-159, doi:10.1111/zsc.12146 (2016).

33 Maynard, B. T., Kerr, L. J., McKiernan, J. M., Jansen, E. S. & Hanna, P. J. Mitochondrial DNA sequence and gene organization in the Australian blacklip abalone *Haliotis rubra* (Leach). *Mar. Biotechnol.* **7**, 645-658, doi:10.1007/s10126-005-0013-z (2005).
